# Synthesis and characterization of nitrogen-doped-MWCNT@cobalt oxide for nerve agent simulant detection

**DOI:** 10.1038/s41598-024-56354-1

**Published:** 2024-05-21

**Authors:** Sanjeeb Lama, Hyeong-Seon Choi, Sivalingam Ramesh, Young Jun Lee, Joo Hyung Kim

**Affiliations:** 1 Laboratory of Intelligent Devices and Thermal Control, Department of Mechanical Engineering, Inha University, Incheon, 22212 South Korea; 2 Department of Mechanical, Robotics and Energy Engineering, Dongguk University, Seoul, South Korea

**Keywords:** Nerve agents, Dimethyl methyl phosphonate, Hydrothermal process, Cobalt oxide, Carbon nanotube, Carbon nanotubes and fullerenes, Nanoparticles, Synthesis and processing

## Abstract

Organophosphorus nerve agents are toxic compounds that disrupt neuromuscular transmission by inhibiting the neurotransmitter enzyme, acetylcholinesterase, leading to rapid death. A hybrid composite was synthesized using a hydrothermal process for the early detection of dimethyl methyl phosphonate (DMMP), a simulant of the G-series nerve agent, sarin. Quartz crystal microbalance (QCM) and surface acoustic wave (SAW) sensors were used as detectors. Nitrogen-doped multiwalled carbon nanotubes (N-MWCNTs), cobalt oxide (Co_3_O_4_), and N-MWCNT@Co_3_O_4_ were compared to detect DMMP concentrations of 25–150 ppm. At 25 ppm, the differential frequencies (Δ*f*) of the N-MWCNT, Co_3_O_4_, and N-MWCNT@Co_3_O_4_ sensors were 5.8, 2.3, and 99.5 Hz, respectively. The selectivity results revealed a preference for the DMMP rather than potential interference. The coefficients of determination (R^2^) of the N-MWCNT, Co_3_O_4_, and N-MWCNT@Co_3_O_4_ sensors for detecting 25–150 ppm DMMP were 0.983, 0.986, and 0.999, respectively. The response times of the N-MWCNT, Co_3_O_4_, and N-MWCNT@Co_3_O_4_ sensors for detecting 100 ppm DMMP were 25, 27, and 34 s, respectively, while the corresponding recovery times were 85, 105, and 181 s. The repeatability results revealed the reversible adsorption and desorption phenomena for the fixed DMMP concentration of 100 ppm. These unique findings show that synthesized materials can be used to detect organophosphorus nerve agents.

## Introduction

**Figure 1:**
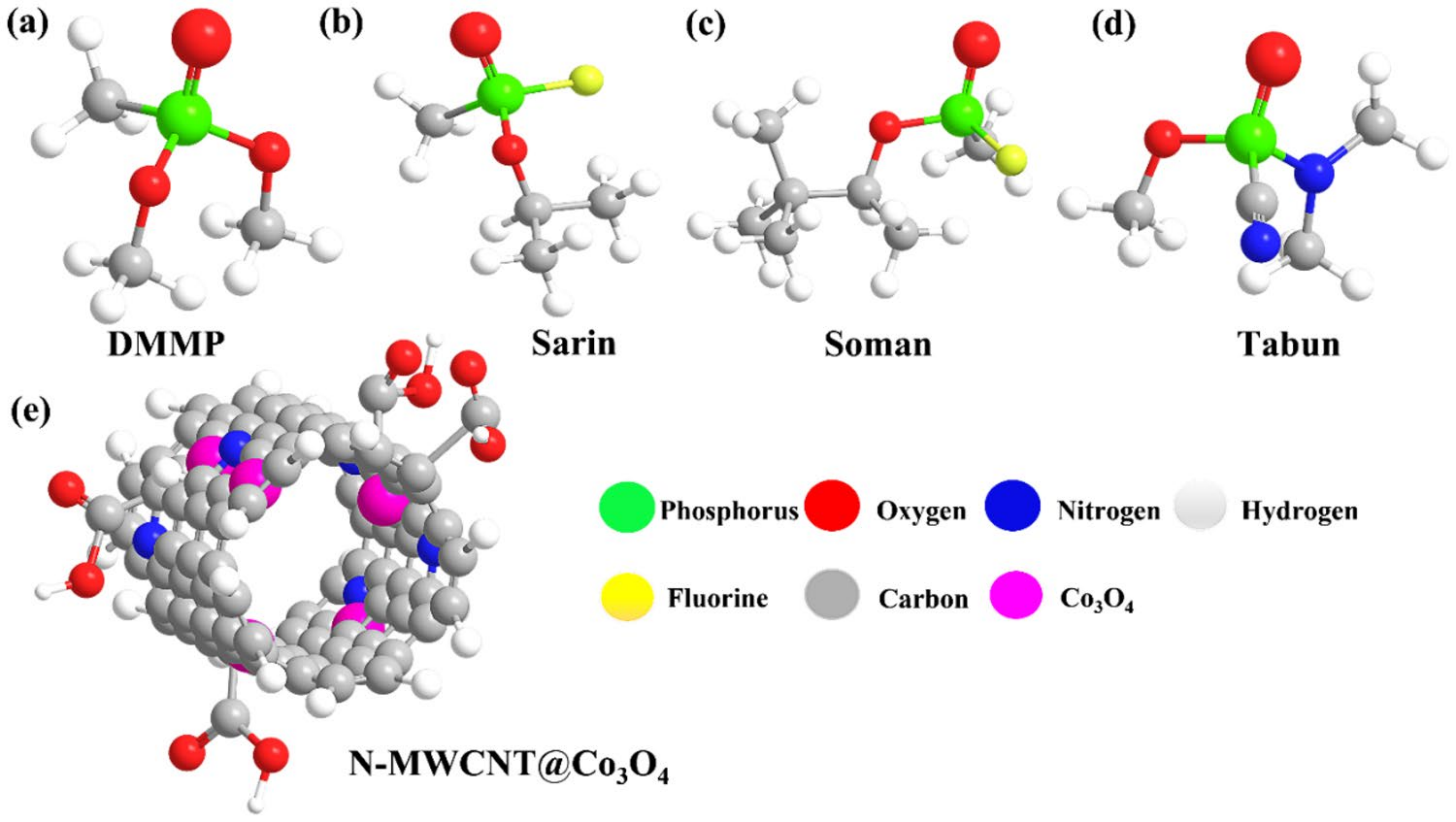
Structural images of the simulant DMMP, sarin (GB series), soman (GD series), and tabun (GA series), and the chemical structure of the hybrid composite N-doped MWCNT(N-MWCNT)@Co_3_O_4_. The figure is not to scale.

Nerve agents (NA) are classified as the most toxic chemical warfare agents that inhibit the activity of acetylcholinesterase, an enzyme responsible for neuromuscular transmission. NA prevents the disintegration of the neurotransmitter acetylcholine into acetate and choline at its esteratic site of the active center, resulting in irreversible acute poisoning caused by the continuous accumulation of the neurotransmitter receptor. When the acetylcholine cannot disintegrate, the muscles are prevented from receiving relaxation signals and are paralyzed. This paralysis can cause severe complications including the heart and muscles for breathing purposes. This may lead to different symptoms within 30 s of exposure and may lead to fatal damage because of asphyxiation within a few minutes depending upon the type and quantity of the nerve agents used. NA were reportedly used in the Iraq–Iran war (1980–1988)^1^, Tokyo subway (1995)^2^, Syria war (2013)^3^, and the assassination of Kim Jong-Nam, the half-brother of the North Korean Leader Kim Jong Un (2017)^4^. These real-world scenarios demonstrate the need for swift responses, such as rapid detection, adequate methodology, preventive measures, and technologies with low power consumption for field monitoring. The use and production of these nerve agents have been strictly monitored and limited by the Organization for the Prohibition of Chemical Weapons (OPCW) because of their deadly nature and high mortality rate. Due to the limitation and high toxicity of sarin nerve agent, dimethyl methyl phosphonate (DMMP), which shares similar chemical structure and much lower toxicity was used as a reference and basis for understanding the interaction between the sensing materials and nerve agent sarin. This paper proposes the use of a quartz crystal microbalance (QCM) and surface acoustic wave sensor (SAW) for the detection of DMMP, which is a simulant of the G-series nerve agent, sarin. Figure 1 presents a 3D picture of the simulant DMMP, sarin (*GB* series), soman (*GD* series), and tabun (*GA* series), as well as the chemical structure of the hybrid composite N-MWCNT@Co_3_O_4_.

QCM sensors are based on bulk acoustic wave sensors whose main principle lies in the piezoelectric quartz crystal, which oscillates at a specified frequency by applying voltage through metal electrodes. QCM sensors have been used in the detection of toxic vapors, volatile organic compounds (VOC), humidity, biomolecules, chiral isomers, contamination in food, bacteria, corrosion, and decay because of their ability to measure changes on the nanogram and microgram scale of the mechanical properties, such as mass, stiffness, and damping. On the other hand, the limitations of QCM sensors lie in their low resonant frequency and their electrode structure in conventional sensors. QCM technique has been used with the several hybrid nanocomposites in the detection of ammonia, humidity, organic acid gas and many more^5–7^. This is because of its simplicity, low cost, high sensitivity and operating functions, compact volume, and measurement possibility at room and elevated temperature which contributed to the selection of this measurement technique.

On the other hand, SAW sensors are based on the changes in the velocity, amplitude, and frequency of the wave generated by the interdigital transducer, traveling through the delay line where adsorption of the analyte by the sensing materials occurs. SAW sensors are used in many applications, such as detecting chemical vapors, biomolecules, proteins, bacteria, pathogens, torque, temperature, and aerosol. The drawbacks of the SAW sensor include low accuracy because of the high-frequency resonator and the reflection of longitudinal waves in the case of liquid measurements. SAW sensors have several characteristics such as high sensitivity, low cost, fast response time, ease of fabrication, and operation at room temperature^8^. Hence, hybrid composite has been used in the SAW sensor for chemical and biological sensing applications.

Since its introduction in 1991 AD, carbon nanotube has been showing huge potential in gas sensors, biosensors, and chemical sensors because of its high thermal and electrical conductivity, high specific surface area, electron transfer ability as well as its adaptability. The geometry, hollow structure, and high surface to volume ratio are crucial for the adsorption of gas molecules. Functionalization of CNTs has been gaining interest as one of the central parts in the research and development, as well as the application of carbon nanotube-based nanocomposite and nanomaterials. Cobalt oxide has been highly investigated because of its spinel structure, mixed oxidation states for swift redox reaction kinetics, low cost, ecofriendly, and ease of preparation^9^. The hybrid composites of Co_3_O_4_@gold/MWCNT/PPy showed a frequency response of 90 Hz for DMMP concentrations of 60 ppm and 98% response and recovery times of 60 and 493 s, respectively^10^. Alali et al. used the electrospinning technique to synthesize the hybrid composite of Co_3_O_4_/CuO heterojunction nanotubes functionalized with hexafluoroisopropanol (HFIP) for the detection of DMMP under visible light irradiation, demonstrating a frequency response of 8.8 *R*_*g*_/*R*_*a*_ at 90 °C, indicating the formation of a hydrogen bond between HFIP and DMMP^11^. MWCNT functionalized with p-hexafluoroisopropanol phenyl showed an insertion loss of 0.35 dB for 20 ppm DMMP in the SAW sensor^12^. Wang et al.^13^ used single-walled carbon nanotubes to detect DMMP that showed a strong and rapid response and recovery and good reproducibility under a density of 30–40 tubes µm^−2^. Furthermore, carbon nanotubes and transition metal oxides like cobalt oxide have been explored in the different fields such as wastewater treatment, electromagnetic wave absorption, strain sensors, lithium batteries, and antimicrobial activity^14–18^. However, to the best of our knowledge, there are limited studies that have focused on the synthesis of N-MWCNT@Co_3_O_4_ and its comparison with the individual N-doped MWCNT (N-MWCNT) and Co_3_O_4_ for the detection of nerve agent simulant, DMMP. In addition, sensing mechanism based on multiple active sites, formation of hydrogen bonds, and interaction with CNT has been discussed to elucidate the sensing mechanism of N-MWCNT@Co_3_O_4_ hybrid composite with the DMMP.

N-MWCNT, Co_3_O_4_, and N-MWCNT@Co_3_O_4_ were synthesized using a hydrothermal process. The sensing materials were characterized by Fourier-transform infrared (FTIR) spectroscopy, powder X-ray diffraction (XRD), X-ray photoelectron spectroscopy (XPS), scanning electron microscopy (SEM), and transmission electron microscopy (TEM). The sensing materials were coated onto the QCM and SAW sensors to detect DMMP concentrations ranging from 25 to 150 ppm. The Δ*f*, selectivity, linearity, repeatability, and response and recovery times were obtained. The sensing mechanisms for the detection of DMMP by the N-MWCNT@Co_3_O_4_ hybrid composite are discussed. The enhanced detection can be attributed to the multiple active sites for the adsorption of DMMP, such as electron transfer from the conduction band that increase or decrease the concentration of holes (charger carrier) as well as ability of Co^3+^ to bind with oxygen atoms of the phosphoryl group in Bronsted acid sites and Lewis acid sites for Co_3_O_4_, formation of hydrogen bonds between carboxylic acid (COOH) group and P=O of DMMP, and intermolecular interaction between the CNTs.

## Results and discussions

### FT-IR data

**Figure 2:**
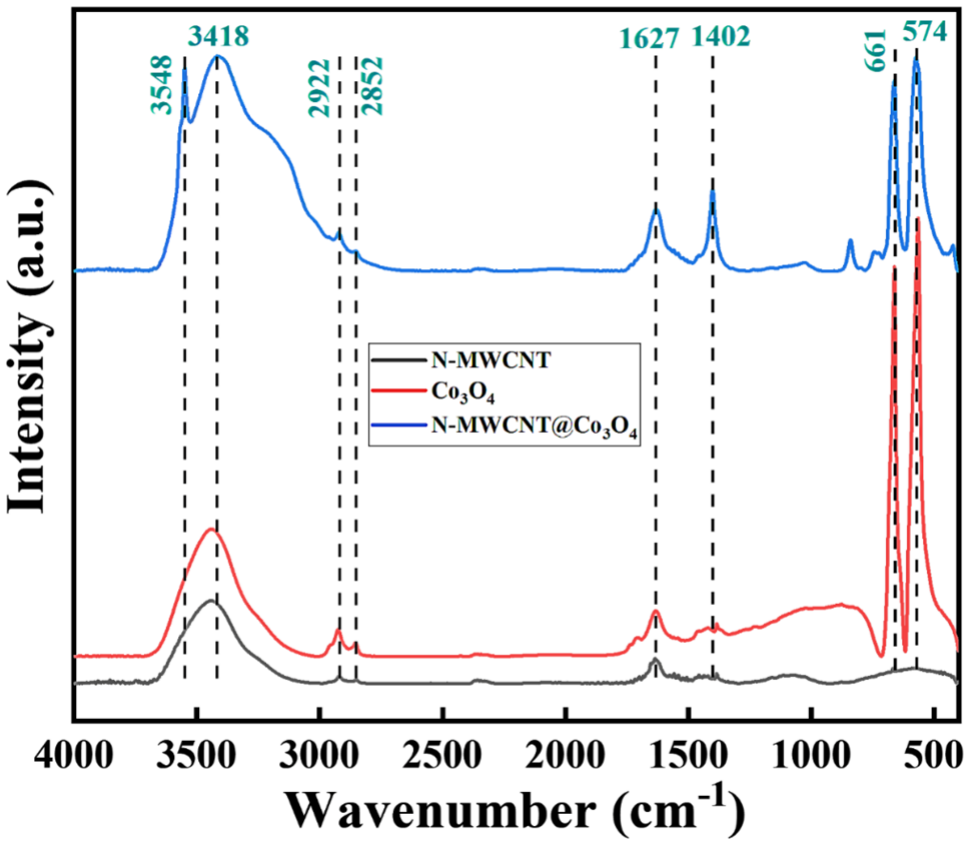
Measured FT-IR spectra of (**a**) Co_3_O_4_, (**b**) N-MWCNT, and (**c**) N-MWCNT@Co_3_O_4_.

Figure 2 presents the FT-IR spectra of the N-MWCNT, Co_3_O_4_, and N-MWCNT@Co_3_O_4_. For Co_3_O_4_, the peaks at 574 and 661 cm^–1^ are characteristic of Co^3+^ in an octahedral site and Co^2+^ in a tetrahedral site in the spinel lattice structure of Co_3_O_4_, respectively^19^. The peak at 1541 cm^–1^ was assigned to the angular deformation of absorbed water molecules. The peaks at 2922 cm^–1^ were attributed to the C–H stretching vibrations^20^. The peak at 3433 cm^–1^ was due to coordinated/entrapped water absorbed from moisture during storage^21^.

For N-MWCNT, the highest peak at 3437 cm^–1^ was attributed to coordinated/entrapped water absorbed from moisture and the –OH stretching vibration. The peak at 1627 cm^–1^ was assigned to the C**=**C stretching vibration of the graphite structure in the MWCNT framework^22^. The peaks at 2852 cm^–1^ and 2922 cm^–1^ are attributed to –CH_3_– and –CH_2_–, respectively. The small but distinct peak at 2355 cm^–1^ corresponded to an ammonium group. The peaks at 1541 cm^–1^ and 1627 cm^–1^ represent the C**=**C stretching vibration of the aromatic structure^23^. The peak at 1082 cm^–1^ corresponds to the C–O–C stretching band. The peaks at 574 cm^–1^ and 661 cm^–1^ were attributed to the characteristics of benzene rings^24^.

Finally, the hybrid composite of N-MWCNT@Co_3_O_4_ showed peaks at 574–661, 1398–1627, 2357, 2852, 2922, and 3418 cm^–1^, which correspond to Co^3+^ in an octahedral site and Co^2+^ in a tetrahedral site, C**=**C stretching vibration of the aromatic structure, ammonium group, –CH_3_–, –CH_2_–, and –OH stretching vibration.

### XRD data

**Figure 3:**
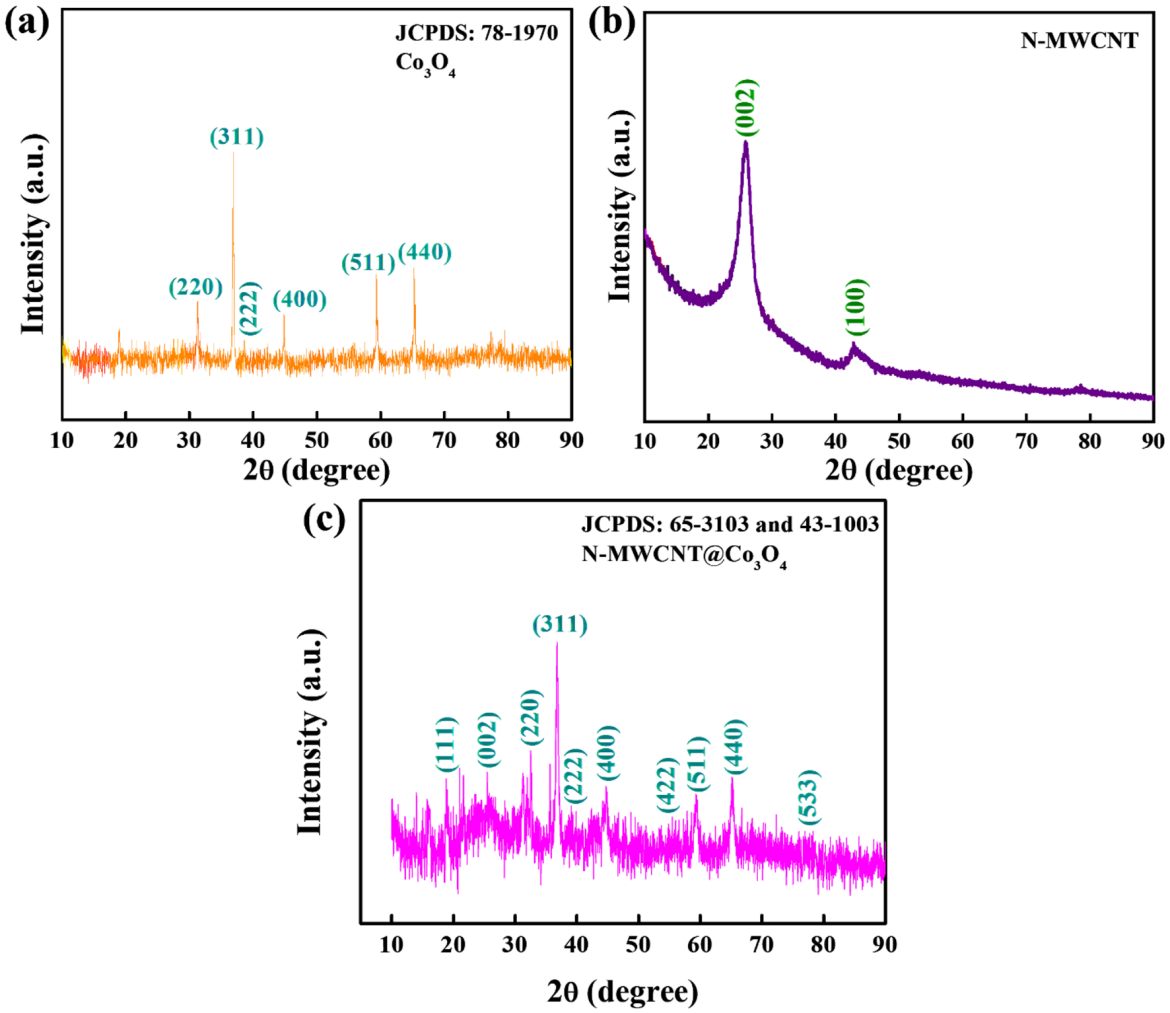
Measured XRD patterns of (**a**) Co_3_O_4_, (**b**) N-MWCNT, and (**c**) N-MWCNT@Co_3_O_4_.

The XRD results of the prepared materials are illustrated in Fig. 3. The XRD peaks at 31.3°, 36.9°, 38.6°, 44.8°, 59.4°, and 65.2° 2θ were indexed to the (220), (311), (222), (400), (511), and (440) planes, respectively (JCPDS file No. 78-1970), representing the face-centered cubic phase Co_3_O_4_ crystalline structure^25^. For the N-MWCNT, the peak at 25.6° 2θ was indexed to the 002 plane of the MWCNTs^26–28^. For the hybrid composite of N-MWCNT@Co_3_O_4_, the peaks at 18.8°, 32.5°, 36.8°, 39.1°, 44.7°, 55.5°, 59.3°, 65.2°, and 76.9° 2*θ* are attributed to planes (111), (220), (311), (222), (400), (422), (511), (440), and (533), respectively, that represent the face-centered cubic phase of the Co_3_O_4_ (JCPDS file No. 65-3103 and JCPDS file no. 43-1003)^26,29^. The inclusion of MWCNTs can be confirmed at 25.5° 2θ, which was indexed to the (002) plane. Also, the hybrid composite was confirmed by XPS and SEM–EDX.

### XPS

X-ray photoelectron spectroscopy was conducted to examine the elemental composition of the synthesized N-MWCNT, Co_3_O_4_, and N-MWCNT@Co_3_O_4_. In the case of N-MWCNT, XPS analysis revealed the presence of C, O, and N (Fig. S1). Table 1 lists the atomic percentage of each element in the synthesized materials from XPS. The atomic percentage of C, O, and N in the N-MWCNT were 96.10%, 2.46%, and 1.44%, respectively. XPS analysis confirmed the presence of C, O, and N in the as-prepared N-MWCNTs.

**Table 1:** Chemical composition of the synthesized materials evaluated using XPS.

Element	at.%		
	N-MWCNT	Co_3_O_4_	N-MWCNT@Co_3_O_4_
Co	–	32.54	7.14
C	96.10	10.93	74.69
O	2.46	53.95	11.89
N	1.44	2.58	3.13

XPS revealed Co, O, C, and N in Co_3_O_4_ (Fig. S2). The Co 2*p*_1/2_ and 2*p*_3/2_ peaks were observed at 794.4 eV and 779.5 eV, respectively (Fig. S2b). The energy difference between 2*p*_1/2_ and 2*p*_3/2_ was 14.9 eV. By the addition of urea during the synthesis process, nitrogen was introduced in the Co_3_O_4_. The atomic percentages of Co, C, O, and N were 32.54%, 10.93%, 53.95%, and 2.58%, respectively (Table 1). Hence, XPS analysis confirmed the inclusion of Co, C, O, and N in the as-prepared Co_3_O_4_.

**Figure 4:**
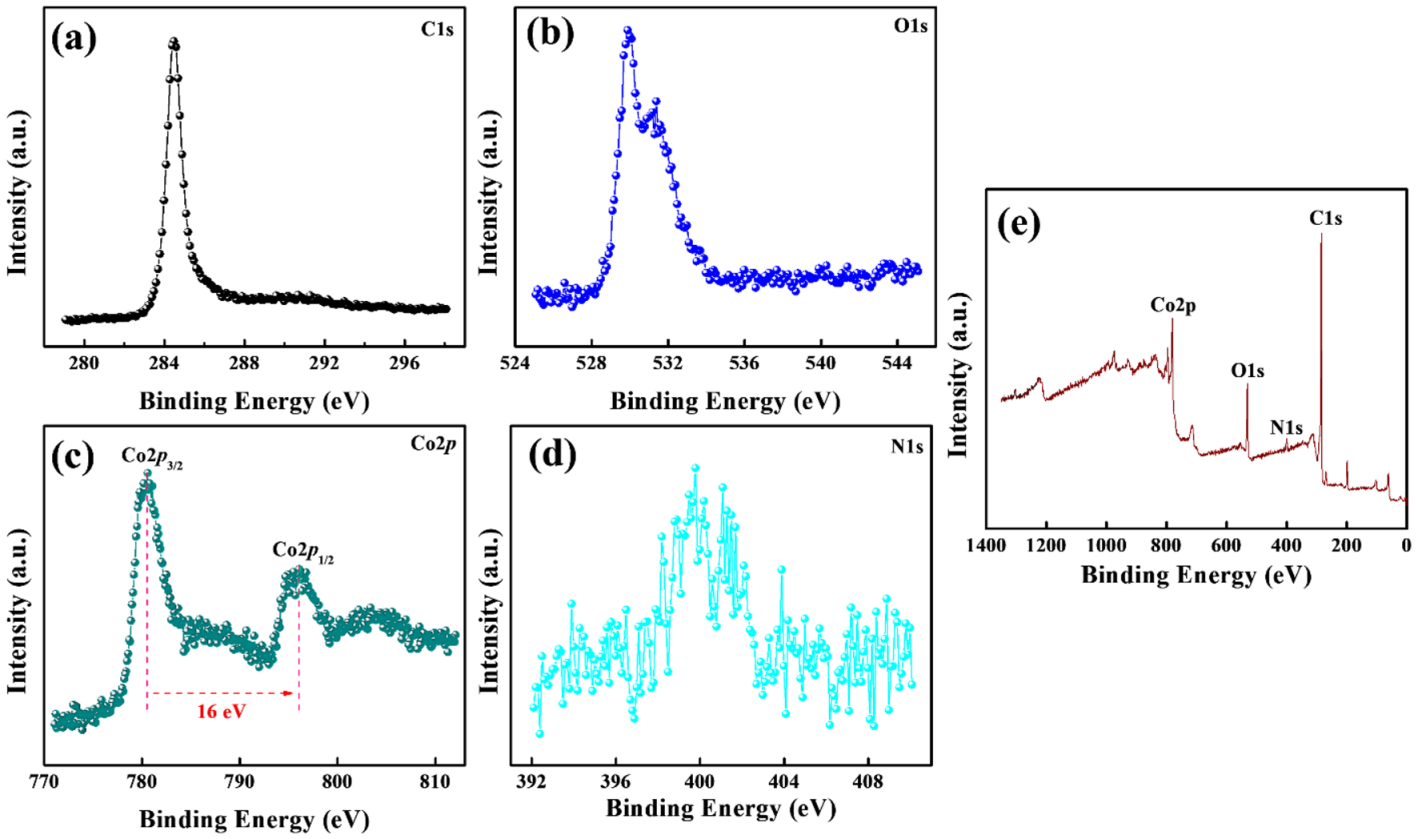
XPS analysis of N-MWCNT@Co_3_O_4_. (**a**) C1s, (**b**) O1s, (**c**) Co2p, (**d**) N1s, and (**e**) survey spectrum.

For N-MWCNT@Co_3_O_4_, XPS revealed the presence of Co, C, O, N, and Cl (see Fig. 4). The atomic percentage of Co, C, O, and N were 7.14%, 74.69%, 11.89%, and 3.13%, respectively. The 2*p*_1/2_ (796.1 eV) and 2*p*_3/2_ (780.6 eV) were characteristic of Co 2*p* (Fig. 4c). The energy difference between 2*p*_1/2_ and 2*p*_3/2_ was 15.5 eV. Hence, XPS provided important evidence confirming the presence of N-MWCNT and Co_3_O_4_ in the hybrid N-MWCNT@Co_3_O_4_ composite.

### SEM data

Figure S3a–f shows the surface morphology of N-MWCNT investigated by field emission SEM (FE-SEM). The fibrils were bundled together and formed an amorphous structure. At high magnification, the fibrils appear to have a coarse structure (Fig. S3f). The estimated diameter of the fibrils was about 20–40 nm. The fibrils appeared white or gray when viewed from the naked eye. Therefore, the noodle-like structure of the N-MWCNT formed the porous structure accompanied by the tangled cross-linked fibrils that offer a large surface area for the adsorption/desorption kinetics.

Figure S4a–f reveals the surface morphology of Co_3_O_4_ at different magnifications. The small semi-circular and semi-rectangular sheet-like structures were stacked on each other, forming a hummock-like structure in Fig. S4f. The surface morphology appeared porous (indicated by the circle), which can provide accessible surface area during the adsorption or desorption. The morphology was bundled or agglomerated to form a pine needle-like structure in Fig. S4d.

**Figure 5:**
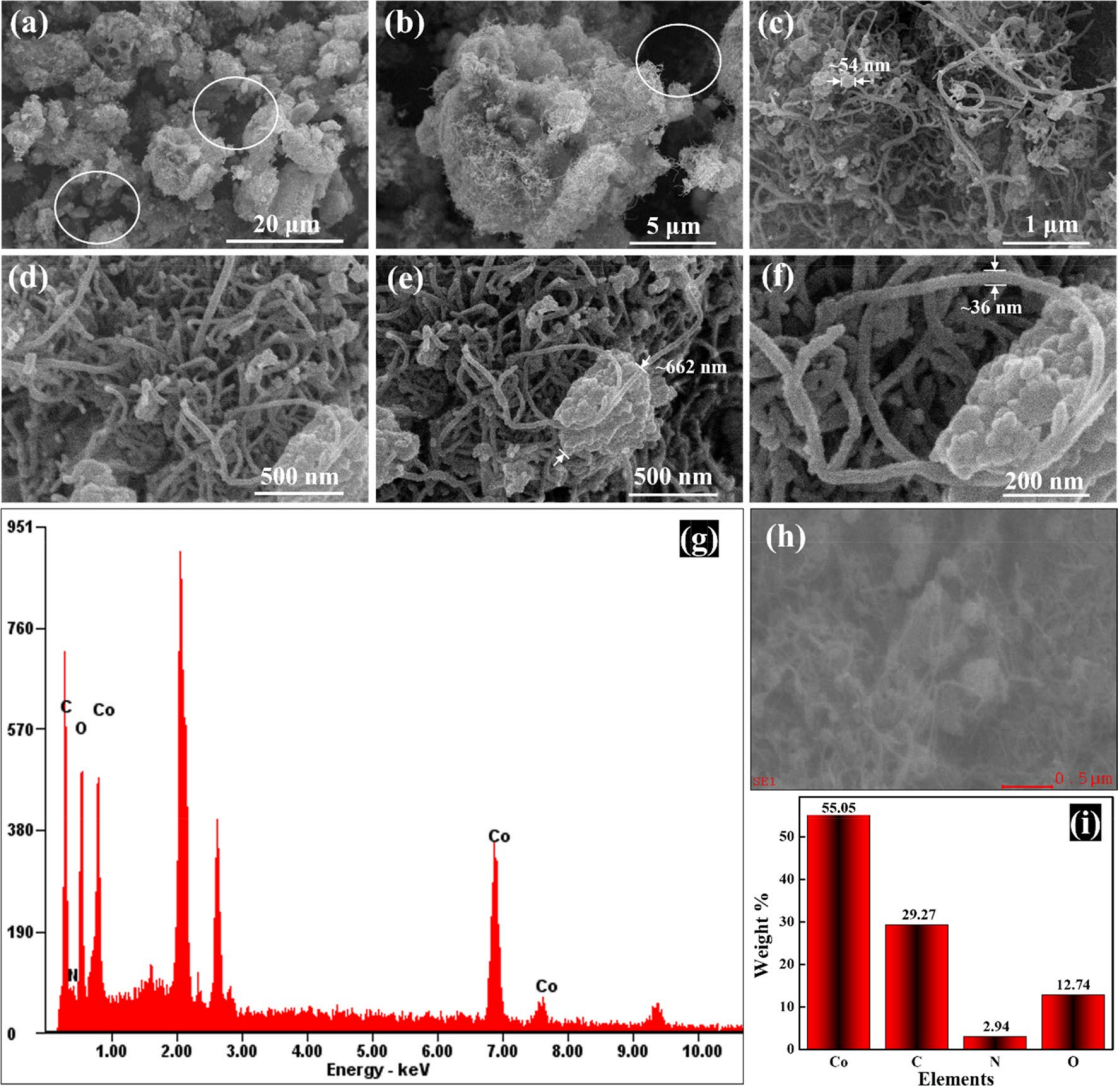
(**a**–**f**) Measured FE-SEM images of the N-MWCNT@Co_3_O_4_ at different magnifications, (**g**–**i**) EDS profile of the N-MWCNT@Co_3_O_4_, and the respective weight percentage of each element.

Figure 5a–f shows the surface morphology of the hybrid composite N-MWCNT@Co_3_O_4_. Co_3_O_4_ was well dispersed within the fibrils of the N-MWCNT (see Fig. 5c). The porous structure (denoted by the circle) of the N-MWCNT@Co_3_O_4_ explains the highly accessible surface area that facilitates the adsorption of the analyte. The hybrid composite appears as a white or gray cotton-ball-like structure. The estimated size of the Co_3_O_4_ in the hybrid composite of N-MWCNT@Co_3_O_4_ was ~ 50–700 nm. In addition, the estimated outer diameter of the MWCNT was about 20–40 nm. SEM–EDS analysis confirmed the presence of Co, C, O, and N in the hybrid composites of N-MWCNT@Co_3_O_4_ (see Fig. 5g–i). The elemental percentage of Co, C, O, and N were 55.05%, 29.27%, 12.74%, and 2.94%, respectively. Hence, Co_3_O_4_ was incorporated into the hybrid N-MWCNT@Co_3_O_4_ composite.

### TEM data

Figure S5a–e presents the TEM results of N-MWCNT at different magnifications, showing MWCNTs with a tubular structure alongside the fibrils. The outer and inner diameters of nanotubes were ~ 22.8 and ~ 6.9 nm, respectively. The estimated inner diameter of the MWCNTs was ~ 5–15 nm. The SAED pattern confirmed the use of CNTs with the (002) plane, indicating the MWCNT interphase boundary (see S5f).

Figure S6a–e presents TEM images of Co_3_O_4_ at different magnifications. The Co_3_O_4_ nanoparticles agglomerated, forming a boomerang-like structure (see Figure S6d). Interestingly, the porous structure or defects (indicated by the circle) were observed on the Co_3_O_4_ nanoparticle surfaces. Hence, it can provide highly accessible areas that increase the adsorption/desorption kinetics. The Co_3_O_4_ nanoparticles constitute the five diffraction rings shown in Fig. S6f, corresponding to the (220), (311), (400), (511), and (440) planes of Co_3_O_4_ nanoparticles. The diffraction rings contained many diffraction spots because 100% uniformity of the particle dispersion was not achieved. Therefore, the architecture of Co_3_O_4_ was a polycrystalline structure^30^. In addition, the SAED pattern confirmed the cubic structure, which agrees with the XRD results^31^.

**Figure 6:**
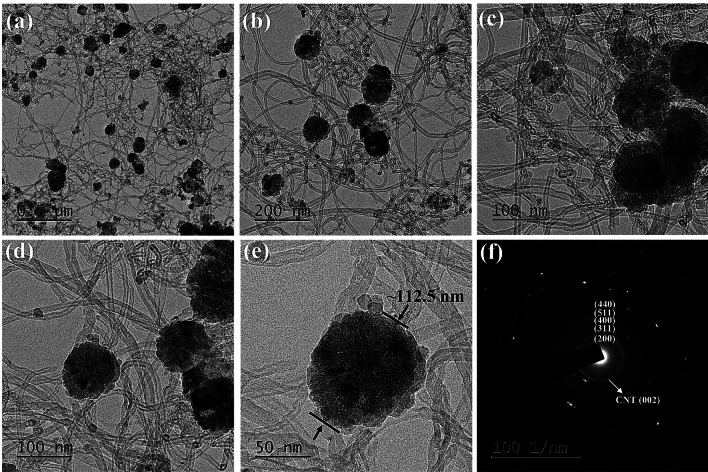
FE-TEM images (**a**–**e**) N-MWCNT@Co_3_O_4_ at different magnifications (**f**) SAED pattern of N-MWCNT@Co_3_O_4_.

Figure 6a–e shows the TEM images of N-MWCNT@Co_3_O_4_ at different magnifications. The Co_3_O_4_ nanoparticles were dispersed onto the N-MWCNT. The estimated size of the Co_3_O_4_ in N-MWCNT@Co_3_O_4_ was ~ 50–700 nm. In the N-MWNCT@Co_3_O_4_, the boundary representing the (002) plane of the carbon nanotubes can be observed vividly in the SAED pattern (see Fig. 6f). On the other hand, the Co_3_O_4_ nanoparticles were not 100% dispersed into the N-MWCNT matrix. Therefore, the diffraction rings in the SAED pattern (see Fig. 6f) constitute the diffraction spots^30^. This provides further evidence of the polycrystalline structure of the N-MWNCT@Co_3_O_4_ composites.

### Experiments with the QCM and SAW sensor

**Figure 7:**
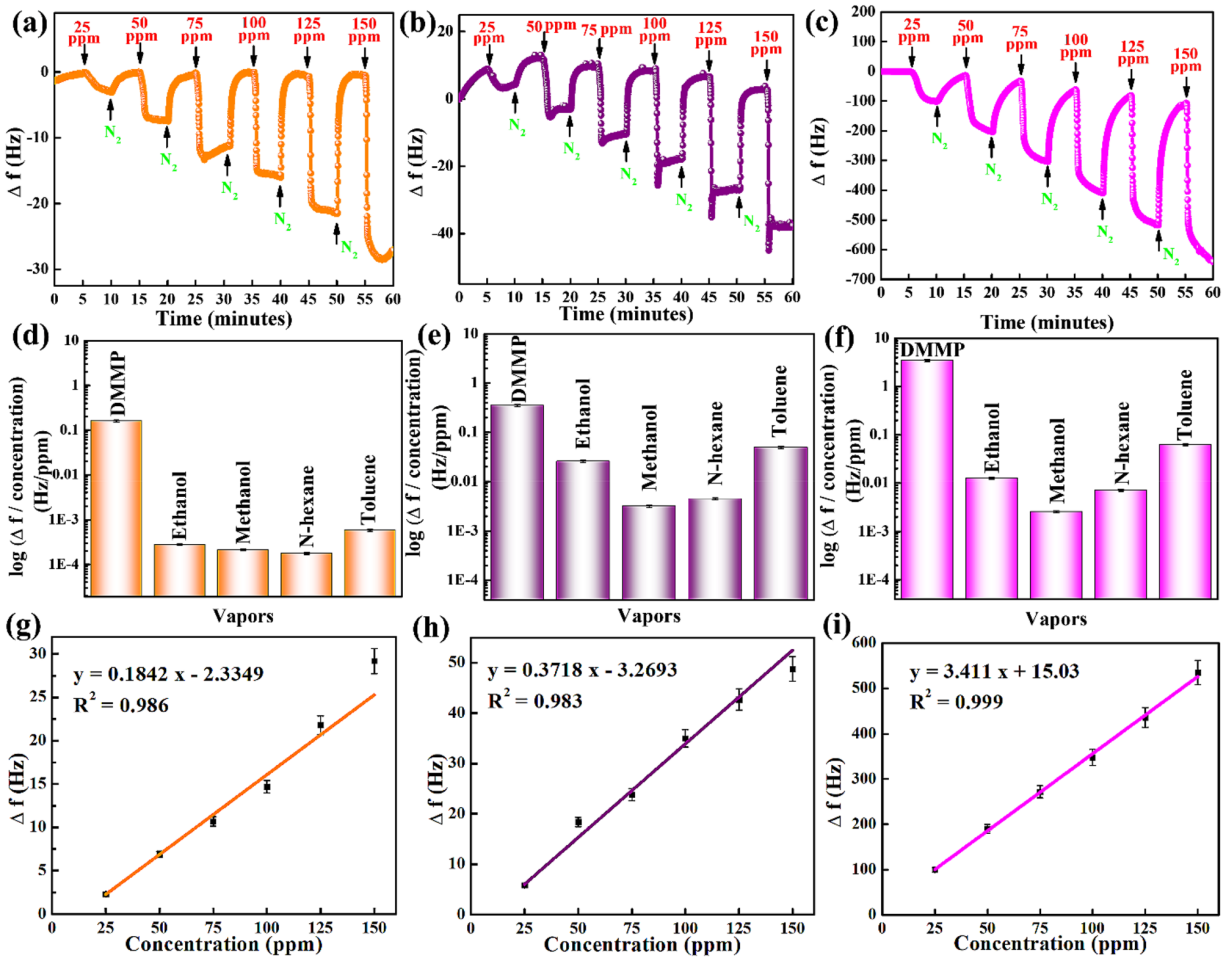
Frequency responses of QCM sensors coated with (**a**) Co_3_O_4_, (**b**) N-MWCNT, (**c**) N-MWCNT@Co_3_O_4_ at DMMP concentrations of 25–150 ppm, selectivity of (**d**) Co_3_O_4_ (**e**) N-MWCNT, (f) N-MWCNT@Co_3_O_4_ at a fixed flow rate (*F*_c_ = 200 sccm and *F*_d_ = 2000 sccm), and the linearity of (**g**) Co_3_O_4_ (**h**) N-MWCNT, (**i**) N-MWCNT@Co_3_O_4_, at six concentrations, increased at 25 ppm intervals.

Figure 7a–c shows the real-time responses of the QCM sensor coated with the N-MWCNT, Co_3_O_4_, and N-MWCNT@Co_3_O_4_ to detect different DMMP concentrations. An immediate change in the frequency shift was observed when DMMP was injected into the detection chamber. The sensing materials were sensitive to DMMP. At a DMMP concentration of 25 ppm, the N-MWCNT, Co_3_O_4_, and N-MWCNT@Co_3_O_4_ displayed Δ*f*′ values of 5.8, 2.3, and 99.5 Hz, respectively. At 150 ppm DMMP, the corresponding Δ*f*′ values were 48.8, 29.2, and 535.3 Hz. Hence, the N-MWCNT@Co_3_O_4_ was approximately 17 times and 11 times better than N-MWCNT at DMMP concentrations of 25 and 150 ppm, respectively. A comparison of the performance with Co_3_O_4_ revealed N-MWCNT@Co_3_O_4_ to show a 43-fold and 18-fold better performance at DMMP concentrations of 25 and 150 ppm, respectively. Hence, the hybrid composite of N-MWCNT@Co_3_O_4_ showed enhanced performance during adsorption. Various mechanisms could play a role in adsorption, demonstrating the synergetic performance of the hybrid composites.

Here, the mass shift Δ*m* is considered the mass of DMMP adsorbed onto the surface of the synthesized N-MWCNT, Co_3_O_4_, and N-MWCNT@Co_3_O_4_ at adsorption. Figure S7 presents the mass of DMMP adsorbed onto the surface of the sensing materials. At 25 ppm, N-MWCNT, Co_3_O_4_, and N-MWCNT@Co_3_O_4_ showed a DMMP intake of 0.10248, 0.04064, and 1.78797 µg/cm^2^, respectively. Hence, N-MWCNT@Co_3_O_4_ absorbed 17.44 times and 43.99 times more than N-MWCNT and Co_3_O_4_, respectively. At 150 ppm DMMP, the masses of DMMP adsorbed by N-MWCNT, Co_3_O_4_, and N-MWCNT@Co_3_O_4_ were 0.86218, 0.51413, and 9.45573 µg/cm^2^, respectively, showing that N-MWCNT@Co_3_O_4_ absorbed 10.96 times and 18.39 times more DMMP than N-MWCNT and Co_3_O_4_, respectively. Hence, the hybrid N-MWCNT@Co_3_O_4_ composites showed enhanced performance while adsorbing DMMP onto the matrix than the individual compounds. The correlation between the frequency shift and the mass of DMMP adsorbed was observed^32^.

**Figure 8:**
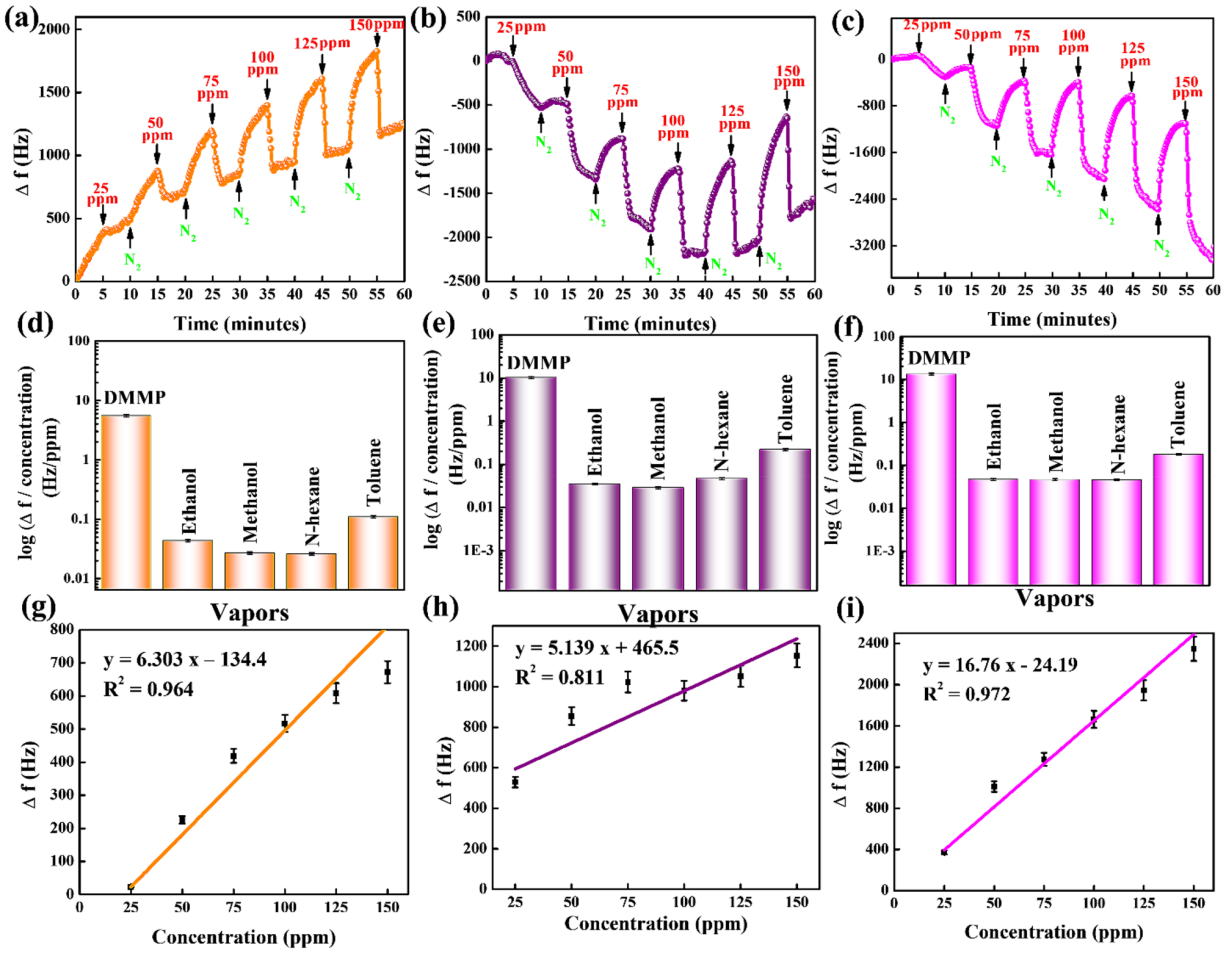
Frequency responses in the SAW sensor coated with (**a**) Co_3_O_4_, (**b**) N-MWCNT, (**c**) N-MWCNT@Co_3_O_4_ at different DMMP concentrations from 25 to 150 ppm, Selectivity of (**d**) Co_3_O_4_ (**e**) N-MWCNT, (**f**) N-MWCNT@Co_3_O_4_ at a fixed flow rate (*F*_c_ = 100 sccm and *F*_d_ = 1000 sccm), and the linearity of (**g**) Co_3_O_4_, (**h**) N-MWCNT, and (**i**) N-MWCNT@Co_3_O_4_, at six different concentrations increased at 25 ppm intervals.

The SAW sensor was coated with the synthesized materials to detect DMMP at different concentrations and provide more evidence that can validate the sensor responses achieved in the QCM sensor. Figure 8a–c presents the real-time responses of the SAW sensor coated with N-MWCNT, Co_3_O_4_, and N-MWCNT@Co_3_O_4_ to detect DMMP concentrations ranging from 25 to 150 ppm. The sensing materials were sensitive to DMMP. At a DMMP concentration of 25 ppm, N-MWCNT, Co_3_O_4_, and N-MWCNT@Co_3_O_4_ displayed Δ*f* values of 529, 22.7, and 372 Hz, respectively. At 150 ppm DMMP, the Δ*f* values for N-MWCNT, Co_3_O_4_, and N-MWCNT@Co_3_O_4_ were 1154, 672, and 2350 Hz, respectively. Hence, N-MWCNT showed a high intake of the DMMP molecules at 25 ppm, while at 150 ppm, N-MWCNT@Co_3_O_4_ showed higher frequency responses than the individual materials. Interestingly, Co_3_O_4_ showed a low-frequency response, possibly due to the weight of cobalt nanoparticles on the SAW sensor (1 mg: 1 mL and 0.5 μL). Although the sensing materials: ethanol ratio was fixed at 1 mg: 1 mL, the weight of the cobalt nanoparticles may have been higher in the SAW sensor because the SAW sensors are highly sensitive toward the mass of the sensing materials.

The QCMs coated with the synthesized N-MWCNT, Co_3_O_4_, and N-MWCNT@Co_3_O_4_ were analyzed to study the linear relationship between Δ*f* and the DMMP concentration (see Fig. 7g–i). N-MWCNT, Co_3_O_4_, and N-MWCNT@Co_3_O_4_ showed R^2^ values of 0.983, 0.986, and 0.999, respectively. Table 2 lists the data used in the calibration curve for the QCM sensor coated with the synthesized materials. The results suggest that the synthesized materials can be promising candidates for practical applications^33^. The experimental results have shown that the N-MWCNT@Co_3_O_4_ coated QCM sensor has exhibited the limit of detection (LOD) of 0.967 ppm.

**Table 2:** Calibration curve data of the QCM sensor coated with N-MWCNT, Co_3_O_4_, and N-MWCNT@Co_3_O_4_.

	N-MWCNT	Co_3_O_4_	N-MWCNT@Co_3_O_4_
Regression equation	y = 0.3718 x − 3.2693	y = 0.1842 x − 2.3349	y = 3.411 x + 15.03
Standard error of the slope	0.0217	0.0099	0.0523
Standard error of the intercept	0.8996	0.3767	2.7869
Coefficient of determination (R^2^)	0.983	0.986	0.999
Number of data points	6	6	6

*y represents the frequency response, and x represents the DMMP concentration.

The SAW coated with the synthesized N-MWCNT, Co_3_O_4_, and N-MWCNT@Co_3_O_4_ were analyzed to examine the linear relationship between Δ*f* and DMMP concentration (see Fig. 8g–i). N-MWCNT, Co_3_O_4_, and N-MWCNT@Co_3_O_4_ presented R^2^ values of 0.811, 0.964, and 0.972, respectively. Table 3 lists the calibration curve data for the SAW sensor coated with synthesized materials.

**Table 3:** Calibration curve data of the SAW sensor coated with N-MWCNT, Co_3_O_4_, and N-MWCNT@Co_3_O_4_.

	N-MWCNT	Co_3_O_4_	N-MWCNT@Co_3_O_4_
Regression equation	y = 5.139 x + 465.5	y = 6.303 x − 134.4	y = 16.76 x − 24.19
Standard error of the slope	0.5426	1.0838	1.2716
Standard error of the intercept	14.5694	84.6619	61.1084
Coefficient of determination (R^2^)	0.811	0.964	0.972
Number of data points	6	6	6

*y represents the frequency response, and x represents the concentration of DMMP.

Selectivity is one of the crucial parameters when determining the response behavior of the sensing materials towards various vapors. The synthesized materials were exposed to various vapors at a fixed flow rate of *F*_c_ = 200 sccm and *F*_d_ = 2000 sccm (see Fig. 7d–f). The calculated DMMP, ethanol, methanol, *n*-hexane, and toluene concentrations at this fixed flow rate were 100, 7525, 17,902, 21,915, and 3522 ppm, respectively. Figure S8 presents the frequency shift of N-MWCNT, Co_3_O_4_, and N-MWCNT@Co_3_O_4_ for several vapors at a fixed flow rate. Interestingly, N-MWCNT showed the least sensitivity towards DMMP (see Fig. S8b). On the other hand, for Co_3_O_4_ and N-MWCNT@Co_3_O_4_, the synthesized materials showed better performance towards the DMMP vapor. Figure 7d–f shows the selectivity test (logarithm of frequency/concentration) of the synthesized materials. Therefore, although there is a high concentration of other vapors, Co_3_O_4_ and N-MWCNT@Co_3_O_4_ will have increased sensitivity towards the DMMP rather than the potential interference. Nevertheless, N-MWCNT might show sensitivity towards other potential interference if exposed to an excessively high concentration of potential interference. It was reported that the selectivity of the gas sensors is related to the dipole moment of the gas molecules^34^. The dipole moment values of DMMP, Ethanol, Methanol, hexane, and Toluene are 3.62D, 1.7D, 1.69D, 0.09, and 0.36D, respectively^35^. It can be observed that the polar molecule DMMP has a higher dipole moment in comparison with the other interfering VOCs, which results in the significant resistance response along with the frequency response. However, together with the dipole moment, several other factors should also be taken into account, such as the charge transfer ability, adsorption energy, and the LUMO (lowest unoccupied molecule orbit) energy values for different gas molecules that accounts for the selectivity^36^.

The synthesized material-coated SAW sensors were exposed to various vapors at fixed flow rates of *F*_c_ = 100 sccm and *F*_d_ = 1000 sccm. Figure 8d–f presents the selectivity test (logarithm of frequency/concentration) of the synthesized materials in terms of frequency and concentration, and Fig. S9a–c shows the selectivity results in terms of frequency shift only. These results show that if there is a high concentration of other vapors, the synthesized materials exhibited an increased frequency shift towards DMMP than potential interference, e.g., at a toluene vapor concentration of 3522 ppm, which was 35-fold higher than that of DMMP. Although the concentration of this potential interference was higher, the frequency response of N-MWCNT@Co_3_O_4_ (13.38 Hz/ppm) towards the DMMP was more than 74 times greater than the frequency response of toluene vapor (0.1799 Hz/ppm)^33^. Thus, the SAW sensor coated with the synthesized materials exhibited excellent frequency responses towards the target CWA and simulant.

The repeatability was examined by subjecting the N-MWCNT, Co_3_O_4_, and N-MWCNT@Co_3_O_4_-coated QCM sensors to 100 ppm DMMP (see Fig. S10a–c). The sensing materials showed reversible adsorption and desorption while detecting and purging DMMP. The response curves did not show significant changes during the adsorption and desorption, but a slight drift was noted. The differences in concentration may have caused the drift because the dilution process cannot control the vapor concentration accurately^37^.

The repeatability of each sensing material was analyzed by calculating the coefficients of variation (*D*) for the repeated responses (n = 4) when exposed to 100 ppm DMMP. The *D* values for the response of the N-MWCNT, Co_3_O_4_, and N-MWCNT@Co_3_O_4_ in the detection of 100 ppm DMMP were calculated using the following equation:

1 $$D = \frac{\delta }{k} \times { 1}00$$

where *δ* is the standard deviation of a set of responses, and *k* is the average response of the QCM sensor coated with the hybrid composites when exposed to a DMMP vapor concentration of 100 ppm. Table 4 lists the calculated *δ*, *k*, and *D* values for N-MWCNT, Co_3_O_4_, and N-MWCNT@Co_3_O_4_. A lower *D* value indicates higher repeatability^38^. N-MWCNT, Co_3_O_4_, and N-MWCNT@Co_3_O_4_ showed *D* values of 15.204%, 1.801%, and 0.553%, respectively.

**Table 4:** Calculated *δ*, *k*, and *D* values.

	N-MWCNT	Co_3_O_4_	N-MWCNT@Co_3_O_4_
Standard deviation, *δ*	5.340 Hz	0.269 Hz	2.071 Hz
Average, *k*	35.122 Hz	14.950 Hz	374.650 Hz
Coefficient of variation, *D*	15.204%	1.801%	0.553%

The repeatability was examined by subjecting the N-MWCNT, Co_3_O_4_, and N-MWCNT@Co_3_O_4_-coated SAW sensors to a fixed concentration of 100 ppm DMMP (see Fig. S11a–c). The sensing material-coated SAW sensor exhibited reversible adsorption and desorption while detecting and purging DMMP, in which DMMP may be attached to the surface of the sensing materials via hydrogen bonding or van der Waals force.

Similar to the QCM sensor, the repeatability of each sensing material in the SAW sensor was evaluated by calculating the *D* values of the repeated responses (n = 4) when exposed to 100 ppm DMMP. The *D* values for the responses of N-MWCNT, Co_3_O_4_, and N-MWCNT@Co_3_O_4_ were evaluated using Eq. (1). Table 5 lists the calculated *δ*, *k*, and *D* values of the given SAW sensor. N-MWCNT, Co_3_O_4_, and N-MWCNT@Co_3_O_4_ had *D* values of 1.464%, 3.131%, and 12.941%, respectively.

**Table 5:** Calculated values of *δ*, *k*, and *D*.

	N-MWCNT	Co_3_O_4_	N-MWCNT@Co_3_O_4_
Standard deviation, *δ*	14.931 Hz	17.241 Hz	173.148 Hz
Average, *k*	1020.115 Hz	550.660 Hz	1338 Hz
Coefficient of variation, *D*	1.464%	3.131%	12.941%

The response and recovery times of the synthesized materials were defined as the time required to reach 90% of the equilibrium after adding DMMP and the time required to reach 10% of the equilibrium when purging with the nitrogen gas. Figure S12a–c presents the response and recovery times of the synthesized materials for a DMMP concentration of 100 ppm. N-MWCNT displayed response and recovery times of 25 s and 85 s, respectively. For Co_3_O_4_, the response and recovery times were 27 s and 105 s, respectively. The N-MWCNT@Co_3_O_4_ showed response and recovery times of 34 s and 181 s, respectively. Thus, the hybrid composites showed longer response and recovery times, possibly because of the large amount of DMMP absorbed and the need to desorb the large amount of DMMP, which agrees with the mass of DMMP accumulated in the sensing materials during the adsorption process mentioned before. Furthermore, longer recovery times indicate that the N-MWCNT@Co_3_O_4_ composite shows enhanced interaction with DMMP molecules compared to individual compounds, increasing the time for desorption of absorbed DMMP molecules. Table 6 compares the response and recovery times of the presented work with the previous literature for detecting DMMP in the QCM sensor.

**Table 6:** Comparison of the response and recovery times with the previous literature for detecting DMMP in the QCM sensor.

Materials	Concentration (ppm)	Response times (s)	Recovery times (s)	References
TiO_2_-SiO_2_-HFIP (S4)	60	< 60	< 60	^39^
Cu-ZSM-5 (5)	40	*T*_80_ < 100	*T*_90_ > 1800	^40^
Co_3_O_4_@gold /MWCNT/ PPy	60	*T*_98_ = 60	*T*_98_ = 493	^10^
PVDF	300	~ 60	~ 60	^41^
NGO@MnO_2_/PPy	75	*T*_90_ = 103	*T*_90_ = 117	^42^
N-MWCNT	100	*T*_90_ = 25	*T*_90_ = 85	This work
Co_3_O_4_		*T*_90_ = 27	*T*_90_ = 105	
N-MWCNT@Co_3_O_4_		*T*_90_ = 34	*T*_90_ = 181	

Figure S13a–c presents the response and recovery times of the synthesized materials for 100 ppm DMMP. N-MWCNT displayed response and recovery times of 66 s and 166 s, respectively. The response and recovery times for Co_3_O_4_ were 46 s and 201 s, respectively. The N-MWCNT@Co_3_O_4_ showed response and recovery times of 50 s and 237 s, respectively. The recovery times of the synthesized materials were low, corresponding to the low desorption rate of DMMP molecules from the SAW surface^43^.

**Figure 9:**
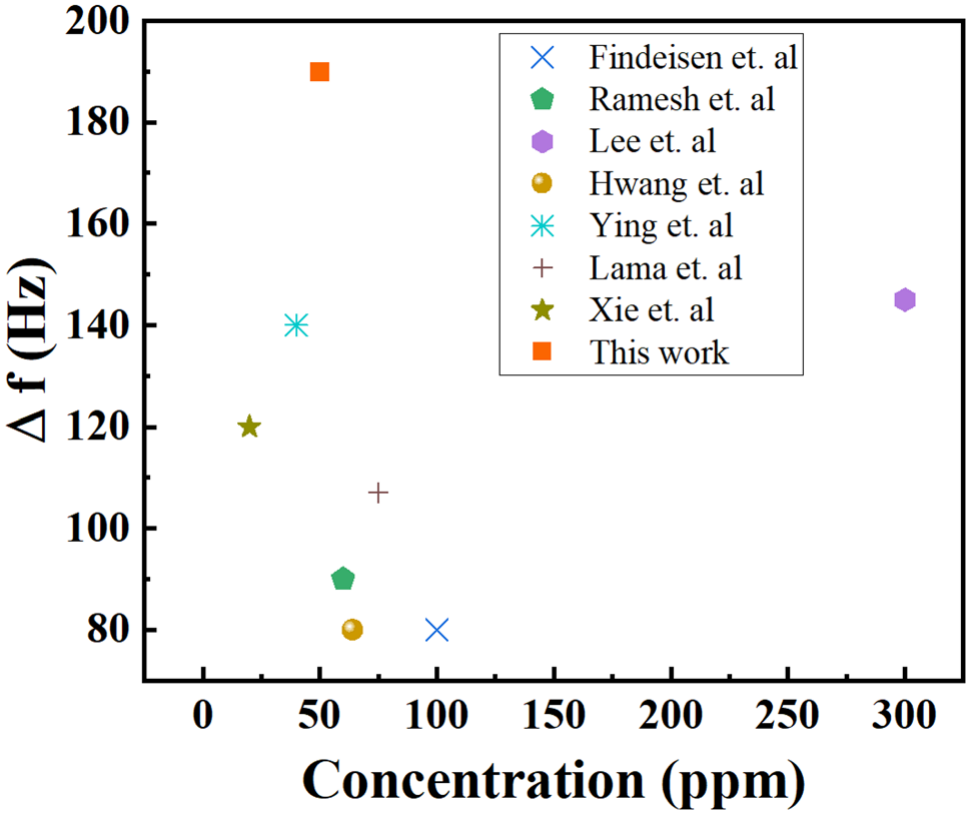
Comparison study of N-MWCNT@Co_3_O_4_ in terms of frequency shift versus concentration with previous studies on QCM sensors.

Many studies have reported using different materials to detect DMMP molecules in a QCM sensor (see Fig. 9)^44,45^. In the present study, the N-MWCNT@Co_3_O_4_ composite showed exceptional performance with Δ*f* of 99.5 Hz and 190.3 Hz at a concentration of 25 and 50 ppm, respectively. Findeisen et al. reported using molecularly imprinted polymers (MIP) as a receptor for detecting DMMP molecules, which showed Δ*f* of ~ 80 Hz at 100 ppm^46^. Polyvinylidene fluoride (PVDF) has been suggested as a sensing material in the QCM-based DMMP sensor that demonstrated Δ*f* of ~ 140 Hz at 300 ppm and response/recovery times of ~ 60/60 s^41^. The use of non-stacked reduced graphene oxide (NSrGO)-hexafluorohydoroxypropanyl benzene (HFHPB) nanosheets that can detect DMMP showing a Δ*f* of ~ 80 Hz at 64 ppm and response/recovery times of ~ 22/27 s^47^. In comparison, our previous work^42^ used NGO@MnO_2_/PPy as sensing materials that showed Δ*f* of 87.4 Hz, 106.7 Hz, and 125.9 Hz in 50, 75, and 100 ppm DMMP vapor, respectively. At 100 ppm, N-MWCNT@Co_3_O_4_ (*k* = 374.65 Hz) performed better than NGO@MnO_2_/PPy (Δ*f* = 125.9 Hz). The Δ*f* of N-MWCNT@Co_3_O_4_ was 2.98 times greater than that of NGO@MnO_2_/PPy. At 50 ppm DMMP vapor, N-MWCNT@Co_3_O_4_ performed 2.18 times better than NGO@MnO_2_/PPy. Hence, it can be concluded that N-MWCNT@Co_3_O_4_ composite can be a promising candidate for detecting DMMP. Table 7 compares the frequency shift (∆*f*) of the presented work with the previous literature for detecting DMMP in the QCM sensor.

**Table 7:** Comparison of the Frequency responses (∆*f*) of the presented work with the previous literature for detecting DMMP in the QCM sensor.

Materials	Concentration (ppm)	∆*f*	References
MIP	100	~ 80	Findeisen et al
Co_3_O_4_@gold /MWCNT/ PPy	60	~ 90	Ramesh et al
PVDF	300	145	Lee et al
HFHPB	64	~ 80	Hwang et al
PVDF	40	140	Ying et al
NGO@MnO_2_/PPy	75	107	Lama et. al
Nanosized ZSM-5	20	120	Xie et al
N-MWCNT@Co_3_O_4_	50	190	This work

### DMMP sensing mechanism

At the room temperature, realization of the gas sensing mechanism usually depends upon the charges transfer between the sensing materials and DMMP. Here, we constructed multiple active sites for adsorption of DMMP. The adsorption of DMMP can take place in three kinds of active sites. Co_3_O_4_, N-MWCNT, and Carboxylic group attached with CNT altogether serve as active sites. (1) **Co**_**3**_**O**_**4**_**:** Under normal air conditions, metal oxides will adsorb the free oxygen molecules (O_2_) from the surrounding air, trapping the electrons from the conduction band and converting them to oxygen species (predominately O −) at medium temperatures^11^. These oxygen species lead to electron transfer from the conduction band to chemisorbed oxygen, resulting in an increase in holes on the surface of the sensing layer and forming a hole accumulation region^11,48^. Herein, when the N-MWCNT@Co_3_O_4_ sensor was exposed to air, the sensor has increased number of holes in air which results in lower resistance. After DMMP is exposed to the sensor, DMMP will be adsorbed onto the N-MWCNT@Co_3_O_4_. This is because DMMP is a strong donor of electrons in which electrons will pass from DMMP to N-MWCNT@Co_3_O_4_^11^. Hence, it results in reduction of holes concentration along with increased resistance^49^. After exposure to air again, the desorption of DMMP takes place which recovers the concentration of holes back to their initial values. Also, some literatures have suggested that the Co^3+^ can bind with the oxygen atoms of the phosphoryl group in Bronsted acid sites and Lewis acid sites during DMMP adsorption^50^.

**Figure 10:**
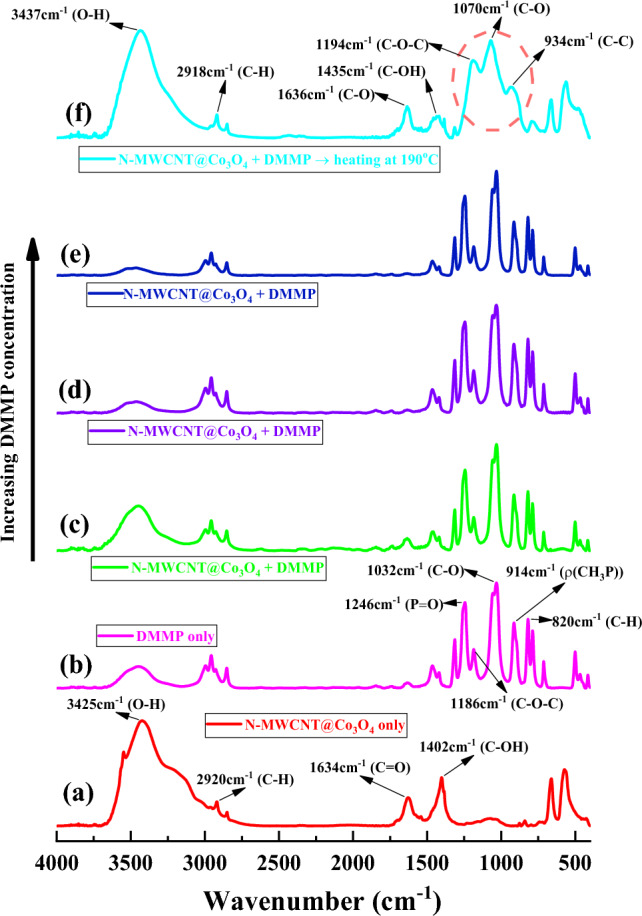
FTIR peaks of (**a**) N-MWCNT@Co_3_O_4_ only, (**b**) DMMP only, (**c**)–(**e**) mixture of N-MWCNT@Co_3_O_4_ and DMMP with increasing concentration of DMMP, and (**f**) mixture of N-MWCNT@Co_3_O_4_ and DMMP after heating at 190 °C for 12 h.

(2)** N-MWCNT:** Various sensing mechanisms have been proposed to explain the sensing mechanism of the carbon nanotube and DMMP. Schroeder et al.^51^ proposed three sensing mechanisms between the carbon nanotube and the analyte: intra-CNT, inter-CNT, and Schottky Barrier. Wang et al.^13^ proposed two sensing mechanisms. The first was direct charge transfer between the deposited SWNTs and DMMP, which reduces the hole density in SWNTs and causes an increase in their electrical resistance^52^. The other was the physisorption of DMMP vapor on SWNT networks. Various active sites available for DMMP sorption exist on the tube surface. The adsorbed DMMP molecules affect electron transport through the SWNT networks, resulting in a change in resistance. In particular, the intercross sites of the SWNT networks may have a significant effect on the sensor behavior^53^. (3)** COOH group**: Furthermore, as shown by FT-IR spectra of N-MWCNT@Co_3_O_4_ while comparing with N-MWCNT and Co_3_O_4_ (see Fig. 2), the intensity of O–H bonding has increased that facilitates the formation of hydrogen bond. It has been reported that the –P–O– of DMMP can react with the O–H group of the carboxylic acid (COOH) via hydrogen bonding^54^. Also, some literature suggest that –COOH surface provides strong interaction with P=O of the adsorbed DMMP to form hydrogen bonds^55^.

Among the several interactions between DMMP and N-MWCNT@Co_3_O_4_ composites, hydrogen bonding interactions have been considered major factors for the room temperature DMMP sensors. FTIR spectroscopy was performed to analyze the adsorption and desorption of DMMP on the N-MWCNT@Co_3_O_4_ hybrid composite. Figure 10 represents the FTIR peaks of (a) N-MWCNT@Co_3_O_4_ only, (b) DMMP only, (c–e) mixture of N-MWCNT@Co_3_O_4_ and DMMP with increasing concentration of DMMP, and (f) mixture of N-MWCNT@Co_3_O_4_ and DMMP after heating at 190 °C for 12 h. It is clearly evident that the P=O of DMMP reacts with the O–H group of the N-MWCNT@Co_3_O_4_ that forms hydrogen bonding which resulted in the decrement of the O–H peak at wavenumber 3425 cm^–1^ as the concentration of DMMP increases. In Fig. 10c, the addition of lowest concentration of DMMP onto the sensing materials resulted in slight diminishing of the O–H group as well as the rise of sharp peaks at wavenumber 934–1194 cm^–1^. As the concentration of the DMMP increases, O–H group can be seen at almost plateau level (see Fig. 10e) while the sharp peaks from 934 to 1194 cm^–1^ remains almost same (see Fig. 10c–e) although the concentration of the DMMP increases.

Another important observation is that after heating the mixture of N-MWCNT@Co_3_O_4_ and DMMP (equal DMMP concentration with Fig. 10e) at 190 °C for 12 h, the peaks at wavenumber 3425 cm^–1^ (O–H group) of the N-MWCNT@Co_3_O_4_ has returned back to its original position (see Fig. 10f). It means that the N-MWCNT@Co_3_O_4_ and DMMP are bonded with the weak force of attraction called hydrogen bonding or Van der Walls force. This is because heating the mixture resulted in the breakage of the hydrogen bonds between the sensing materials and the target analyte as well as the revival of O–H group of sensing materials.

In addition, it seems that the peak of C–H bonding at wavenumber 2918 cm^–1^ increases as the concentration of DMMP increases. However, the peak at wavenumber 1628 cm^–1^ which represents carbonyl group (C=O) that decreases as the concentration of DMMP increases. It indicates that the carbonyl group has participated in the formation of the hydrogen bonding between the sensing materials and target analyte^56^. This is because addition of DMMP causes this peak of N-MWCNT@Co_3_O_4_ to disappear which means that sensing material has formed weak interaction with DMMP causing the peaks to be reduced at base level (see Fig. 10c–e). When heated at 190 °C for 12 h, the peak retains its initial position (see Fig. 10f) which implies that previously sensing materials reacted with DMMP via weak force of attraction and upon heating caused the breakage of this weak force of attraction.

Furthermore, another important observation was the formation of covalent bond at wavenumber 934 cm^–1^ (C–C), 1070 cm^–1^ (C–O), and 1194 cm^–1^ (C–O–C)^57,58^ (orange dotted circle at Fig. 10f). Although we heated the mixture of the N-MWCNT@Co_3_O_4_ and DMMP at 190 °C for 12 h, it showed no sign of breakage which implies that it has formed strong covalent bond that can withstand the temperature of 190 °C for 12 h. Consequently, it requires substantial energy to break such strong covalent bond. Hence, these FTIR spectroscopy results confirms the formation of hydrogen bonding (between P=O of DMMP and O–H of N-MWCNT@Co_3_O_4_) and strong covalent bonds (at wavenumber 934–1194 cm^–1^).

In addition, we conducted the Ultraviolet–visible spectroscopy of the N-MWCNT@Co_3_O_4_ only, DMMP only, and mixture of N-MWCNT@Co_3_O_4_ and DMMP (see Fig. ). It can be observed that a slight blue shift in the absorption peak of DMMP from 282.5 to 281.5 nm and 360 to 359.5 nm when comparing with the mixture of N-MWCNT@Co_3_O_4_S14 and DMMP. When the N-MWCNT@Co_3_O_4_ (powdered—black color) was mixed with DMMP (colorless), after short while it gives distinct cyan-greenish color due to the presence of cobalt oxide^59^ (see Figure S14—optical image). This result indicates that the sensing material shows some absorbance towards the DMMP that emits distinct color during their interaction.

To further prove the sensing mechanism, adsorption capacity (mass of DMMP adsorbed, ∆*m*) and frequency shift (∆*f*) shown by N-MWCNT, Co_3_O_4_, and N-MWCNT@Co_3_O_4_ during the 100 ppm DMMP adsorption process was examined using the QCM technique (see Fig. S15). A correlation between ∆*f* and ∆*m* was observed. At 100 ppm, the mass of DMMP adsorbed by N-MWCNT@Co_3_O_4_ was approximately 9.94 times and 23.67 times higher than N-MWCNT and Co_3_O_4_, respectively. Regarding ∆*f*, N-MWCNT@Co_3_O_4_ showed 10.66 times and 25.06 times higher sensitivity than N-MWCNT and Co_3_O_4_, respectively.

**Figure 11:**
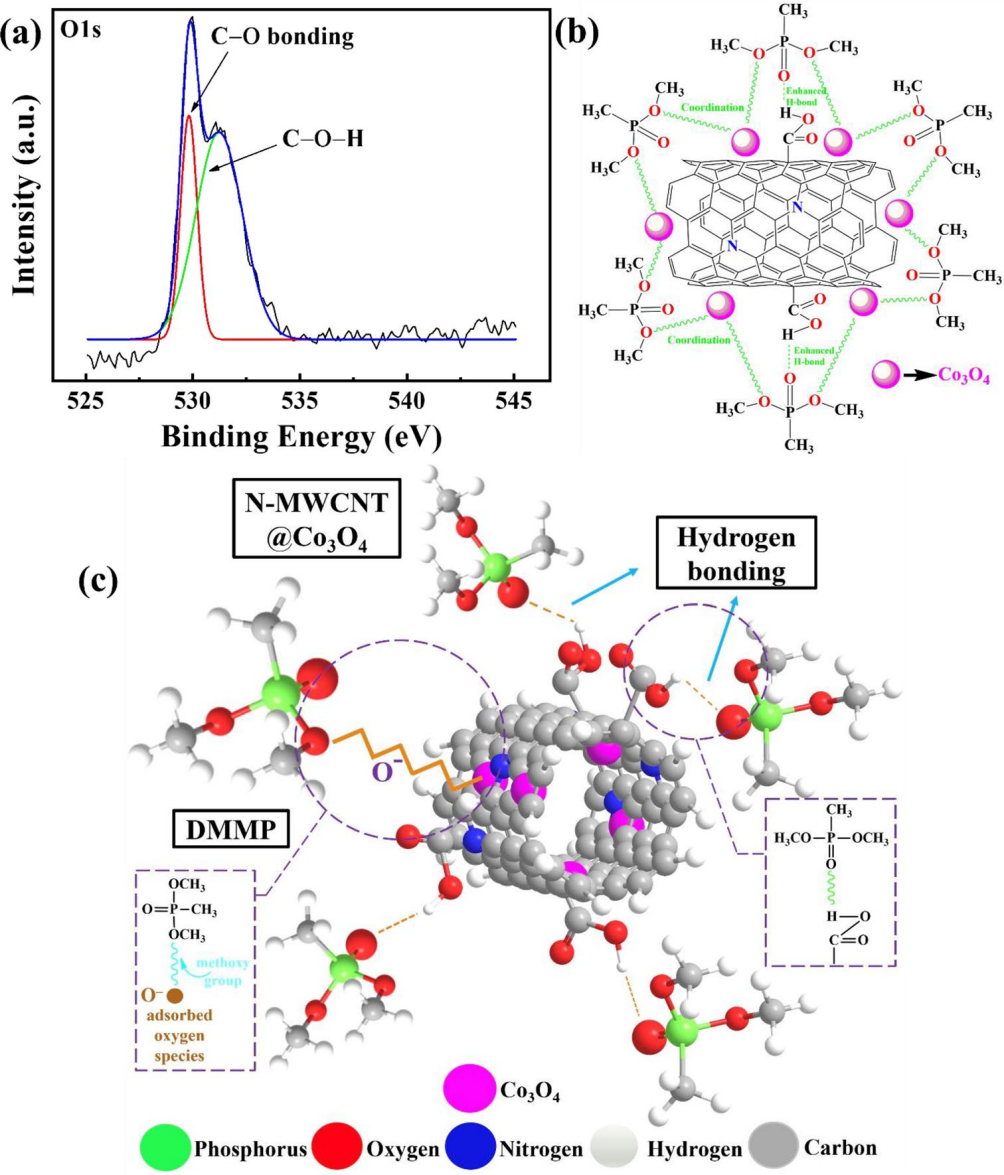
(**a**) Deconvoluted O1s graph of N-MWCNT@Co_3_O_4_, Proposed (**b**) 2D and (**c**) 3D sensing mechanism between the N-MWCNT@Co_3_O_4_ with DMMP. Figure (**b**) and (**c**) are not to scale.

Moreover, it can be observed that the surface of the N-MWCNT@Co_3_O_4_ is covered with the functional groups of –O, and –OH, as confirmed by the deconvoluted XPS (see Fig. 11a)^60^. Also, EDS study confirms the presence the oxygen with weight percentage of 12.74% (Fig. 5i). Hence, as reported in the previous literature, these functional groups tend to form the hydrogen bonding or Van der Walls force^61,62^. Hence, these results also indicate that the sensing materials are bonded with the DMMP via a weak force of attraction called hydrogen bonding or Van der Walls force.

Based on the above discussion, the enhanced sensitivity for DMMP in the N-MWCNT@Co_3_O_4_ sensors is attributed to the construction of multisite for the adsorption of DMMP as well as the synergetic effect between Co_3_O_4_, N-MWCNT, and COOH group. Here, the enhanced DMMP sensing performance by Co_3_O_4_ species was attributed to transfer of electrons in holes (charger carrier) as well as the ability of Co^3+^ to bind with oxygen atoms of phosphoryl group in Bronsted acid sites and Lewis acid sites. Therefore, it is deduced that Co_3_O_4_ species also acts as an active site in this work. Figure 11b,c illustrates the multisite strategy to enhance sensitivity for DMMP adsorption for N-MWCNT@Co_3_O_4_ based sensors.

## Conclusions

The as-prepared materials were synthesized using a hydrothermal process to detect DMMP, a simulant of the nerve agent, sarin, at concentrations ranging from 25 to 150 ppm. The synthesized materials were characterized by FT-IR spectroscopy, XRD, XPS, SEM, and TEM. The frequency response, selectivity, linearity, repeatability, and response and recovery times were obtained on the QCM and SAW sensor to detect the DMMP at 22 ± 2 °C and 25–30% relative humidity. At 25 ppm, N-MWCNT@Co_3_O_4_ showed approximately 17 times and 43 times higher performance than N-MWCNT and Co_3_O_4_, respectively. At 150 ppm DMMP, the intake of the DMMP molecules by N-MWCNT, Co_3_O_4_, and N-MWCNT@Co_3_O_4_ were 0.86218, 0.51413, and 9.45573 µg/cm^2^, respectively. N-MWCNT, Co_3_O_4_, and N-MWCNT@Co_3_O_4_ showed R^2^ values of 0.983, 0.986, and 0.999, respectively. This study provides insight into the ability of the hybrid composite to produce synergetic effects during the adsorption of a nerve agent simulant. Future studies will examine using the as-prepared materials to detect lower DMMP concentrations, mainly at the ppb level.

## Experimental details

### Fabrication of N-MWCNT@ Co_3_O_4_

#### Synthesis of N-MWCNT and Co_3_O_4_

The materials used in synthesizing these hybrid composites are described in the supplementary information. A hydrothermal process was used to synthesize the N-MWCNTs. Briefly, 1 g of MWCNTs was added to 100 mL of distilled water. The mixture was dispersed into a 500 mL beaker. Subsequently, 1.2 g of urea was liquified and added to the mixture. Subsequently, 25 mL of NH_4_OH was poured into the mixture. While performing these processes, the solution was stirred continuously at 95 °C and stirred further at the same temperature for 12 h. The solution was then transferred to the oven at 180 °C for 12 h. After heating, the resulting residue was purified sequentially by water and ethanol. Finally, N-MWCNTs were obtained after purification and heating at 180 °C for 12 h. The N-MWCNTs were kept in an airtight bottle for characterization and experimentation.

For Co_3_O_4_, 1 g of Co (CH_3_COO)_2_·4H_2_O was added to 100 mL of distilled water. Subsequently, 1.2 g of urea was liquified and added to the mixture. The mixture was then transferred to a 500 mL beaker. Subsequently, 25 mL of NH_4_OH was poured into the mixture. A procedure similar to the abovementioned process was applied to obtain the Co_3_O_4_.

#### Synthesis of N-MWCNT@ Co_3_O_4_

**Figure 12:**
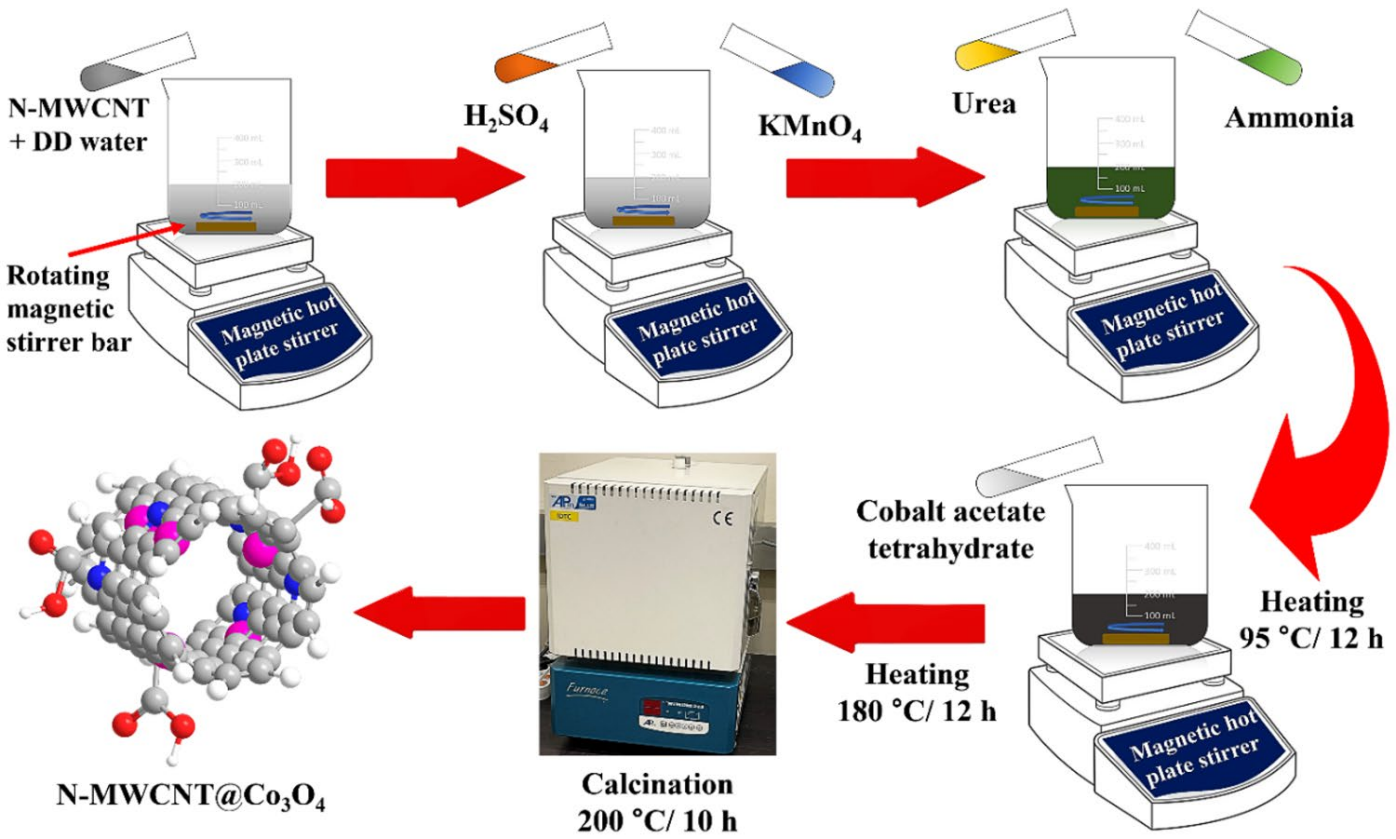
Fabrication process of N-MWCNT@ Co_3_O_4_ composite materials.

**Figure 13:**
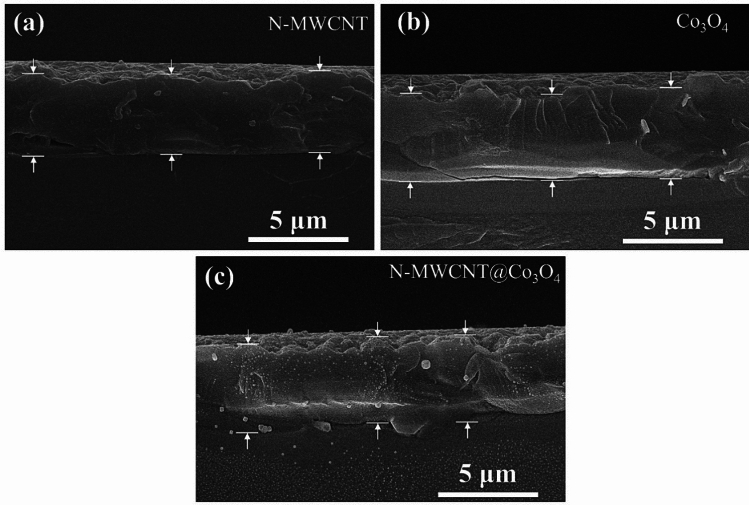
Cross-sectional SEM images of the deposited (**a**) N-doped MWCNT, (**b**) Co_3_O_4_, and (**c**) N-MWCNT@Co_3_O_4_, onto the QCM sensor.

Figure 12 shows the fabrication process of N-MWCNT@ Co_3_O_4_. Briefly, 1 g of MWCNTs was added to 100 mL of distilled water. The mixture was dispersed into a 500 mL beaker. Subsequently, 1.2 g of urea was liquified and added to the mixture, followed by the addition of 0.3 mol of Co (CH_3_COO)_2_·4H_2_O to the mixture. Twenty-five milliliters of NH_4_OH were then poured into the mixture. From this point, the previously mentioned processes were carried out to obtain the hybrid N-MWCNT@Co_3_O_4_ composite. The mean thickness of the deposited N-MWCNT, Co_3_O_4_, and N-MWCNT@Co_3_O_4_ was 4.05063, 4.27848, and 4.02954 µm, respectively, as shown in Fig. 13. The supplementary information explains the target vapor generation, sensor measurement system, and data acquisition, deposition of the sensing materials onto the QCM and SAW sensor, and characterization method.

### Supplementary Information

Supplementary Information.

## Data Availability

The datasets used and/or analyzed during the current study available from the corresponding author on reasonable request.
