# Supplementary material for: Synthesis and characterization of nitrogen-doped-MWCNT@cobalt oxide for nerve agent simulant detection
Source: Sci Rep. 2024 May 21;14:11605. doi: 10.1038/s41598-024-56354-1 (PMC11109131; doi:10.1038/s41598-024-56354-1)
Supplement: Supplementary file 1 — Supplementary Information. [file 41598_2024_56354_MOESM1_ESM.pdf]

(Supplementary information)

Synthesis and characterization of nitrogen-doped-MWCNT@cobalt oxide for nerve agent simulant detection

Sanjeeb Lama [1], Hyeong-Seon Choi [1], Sivalingam Ramesh [1, 2], Young Jun Lee [1, *], Joo Hyung Kim [1, *]

[1] Laboratory of Intelligent Devices and Thermal Control, Department of Mechanical Engineering, Inha University, Incheon 22212, Korea; sanjeeblama132@gmail.com (S.L.); skyman887@inha.edu (H.-S.C.); 318169@inha.ac.kr (Y.-J.L.)

[2] Department of Mechanical, Robotics and Energy Engineering, Dongguk University, Seoul, South Korea; sivaramesh1064@gmail.com (S.R.)

** Corresponding author - joohyung.kim@inha.ac.kr*

Figure S1. XPS analysis of N-MWCNT. (a) C1s, (b) O1s, and (c) N1s, and (d) survey spectrum.5
Figure S2. XPS analysis of Co_3O_4. (a) O1s, (b) Co2p, (c) C1s, and (d) N1s, and (e) survey spectrum. 6
Figure S3. (a-f) FE-SEM images of the N-MWCNT at different magnifications. 7
Figure S4. (a-f) FE-SEM images of Co_3O_4 at different magnifications. 8
Figure S5. (a-e) FE-TEM images of N-MWCNT at different magnifications (f) SAED pattern of N-MWCNT. 9
Figure S6. (a-e) FE-TEM images of Co_3O_4 at different magnifications and (f) SAED pattern of Co_3O_4. 10
Figure S7. Mass of DMMP adsorbed by (a) Co_3O_4 (b) N-MWCNT, and (c) N-MWCNT@Co_3O_4 for DMMP concentrations ranging from 25 to 150 ppm. 11
Figure S8. Selectivity of QCM sensors coated with (a) Co_3O_4, (b) N-MWCNT, (c) N-MWCNT@Co_3O_4 at a fixed flow rate (F_c = 200 sccm and F_d = 2000 sccm). 12
Figure S9. Selectivity of SAW sensors coated with (a) Co_3O_4, (b) N-MWCNT, (c) N-MWCNT@Co_3O_4 at a fixed flow rate (F_c = 100 sccm and F_d = 1000 sccm). 13
Figure S10. Repeatability of QCM sensors coated with (a) Co_3O_4, (b) N-MWCNT, and (c) N-MWCNT@Co_3O_4 at a fixed concentration of 100 ppm DMMP vapor. 14
Figure S11. Repeatability of SAW sensors coated with (a) Co_3O_4 (b) N-MWCNT, and (c) N-MWCNT@Co_3O_4 at a fixed DMMP vapor concentration of 100 ppm. 15
Figure S12. Response and recovery times of QCM sensors coated with (a) Co_3O_4, (b) N-MWCNT, and (c) N-MWCNT@Co_3O_4 at a fixed DMMP vapor concentration of 100 ppm. 16
Figure S13. Response and recovery times of SAW sensors coated with (a) Co_3O_4, (b) N-MWCNT, and (c) N-MWCNT@Co_3O_4 at a fixed DMMP vapor concentration of 100 ppm. 17
Figure S14. Absorbance spectra of N-MWCNT@Co_3O_4 only, DMMP only, and mixture of N-MWCNT@Co_3O_4 and DMMP. 18
Figure S15. Comparison of frequency shift (Δf) and mass shift (Δm) of Co_3O_4, N-MWCNT, and N-MWCNT@Co_3O_4 at a fixed DMMP vapor concentration of 100 ppm. 19

Materials

Multiwalled carbon nanotubes (MWCNT) (20–40 nm), urea, cobalt acetate tetrahydrate [Co $(CH_3COO)_2 \cdot 4H_2O$], potassium permanganate ($KMnO_4$), Hydrochloric acid (HCl), sulfuric acid (H_2SO_4), ammonium hydroxide (NH_4OH), hydrogen peroxide (H_2O_2), dimethyl methyl phosphonate (DMMP), ethanol (C_2H_5OH), methanol (CH_3OH), n-hexane (C_6H_{14}), and toluene (C_7H_8), were acquired from Sigma–Aldrich, Seoul, Korea.

Target vapor generation, sensor measurement system, and data acquisition

Details of target vapor generation, sensor measurement system, and data acquisition are reported elsewhere [1]. The QCM and SAW sensor used in the experiment is reported elsewhere [1].

Deposition of the sensing materials onto the QCM and SAW sensor

The bare QCM sensor was soaked with a piranha solution (30% H_2O_2: 99% H_2SO_4; 1:3 *v*/*v*) for 30 min. The treated QCM sensor was washed sequentially with pure water and ethanol. The QCM sensor was kept in an oven at 60 °C for one hour.

The N-MWCNT, Co_3O_4, and N-MWCNT@Co_3O_4 were stirred with ethanol in a 10 mg:1 mL ratio. The resulting solutions were ultrasonicated for three hours. Subsequently, 15 µL of the acquired suspension was drop-coated carefully at the center of the treated QCM sensor. The drop-coated suspension was allowed to volatilize naturally. The sensing materials deposited on the QCM sensors were heated for one hour at 60 °C in an oven. Finally, the QCM sensors were cooled to 22 ± 2 °C and used as received.

For the SAW sensor, N-MWCNT, Co_3O_4, and N-MWCNT@Co_3O_4 were stirred with ethanol at a 1 mg:1 mL ratio. The resulting solutions were ultrasonicated for three hours. Subsequently, 0.5 µL of the acquired suspension was drop-coated carefully onto the delay line of the SAW sensor. The drop-coated suspension was allowed to volatilize naturally. The sensing materials deposited on SAW sensors were heated for one hour at 60 °C in an oven. The SAW sensors were cooled to 22 ± 2 °C and used as received.

1.1. Characterization methods

Fourier transform infrared (FTIR) spectroscopy of the hybrid composites was conducted on a Vertex 80 v FT-IR Spectrometer (Bruker, Billerica, MA, USA). X-ray diffraction (XRD) of the hybrid composites was performed using an X'pert PRO MRD diffractometer (Philips/Panalytical, Malvern, UK). The surface morphologies of the hybrid composites were analyzed by FE-SEM—S-4300SE (Hitachi, Tokyo, Japan). The morphological structure, thickness, and diameter of the hybrid composites were investigated by FE-TEM—JEM-2100F (Jeol, Akishima, Japan). X-ray photoelectron spectroscopy (XPS, K-Alpha, Thermo Fisher Scientific, Waltham, MA, USA) was used to analyze the elemental composition of the composite materials. The QCM controller system (QCM200, Stanford Research System, Sunnyvale, CA, USA) was used to measure the changes in frequency during adsorption and desorption.

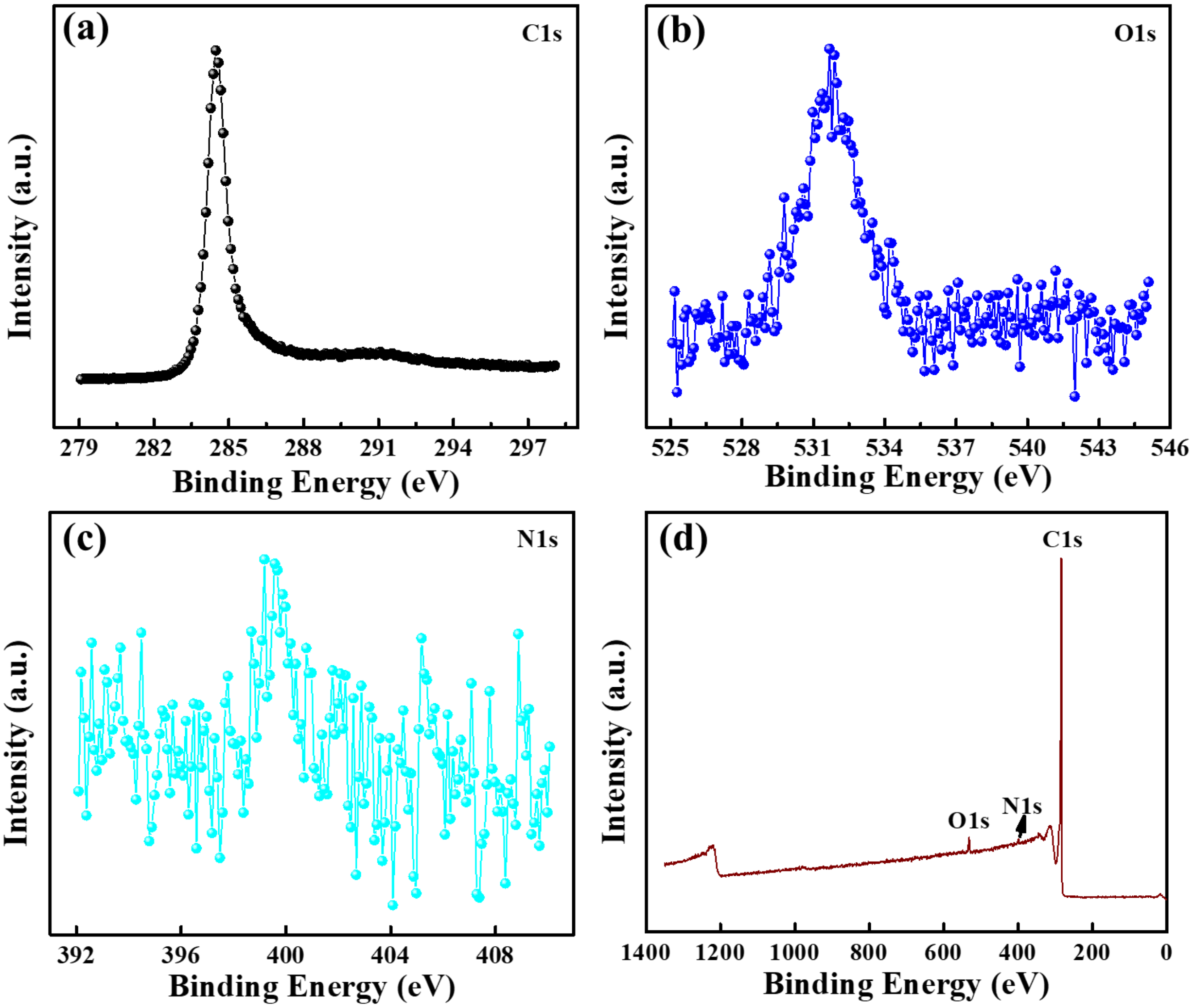

Figure S1. XPS analysis of N-MWCNT. (a) C1s, (b) O1s, and (c) N1s, and (d) survey spectrum.

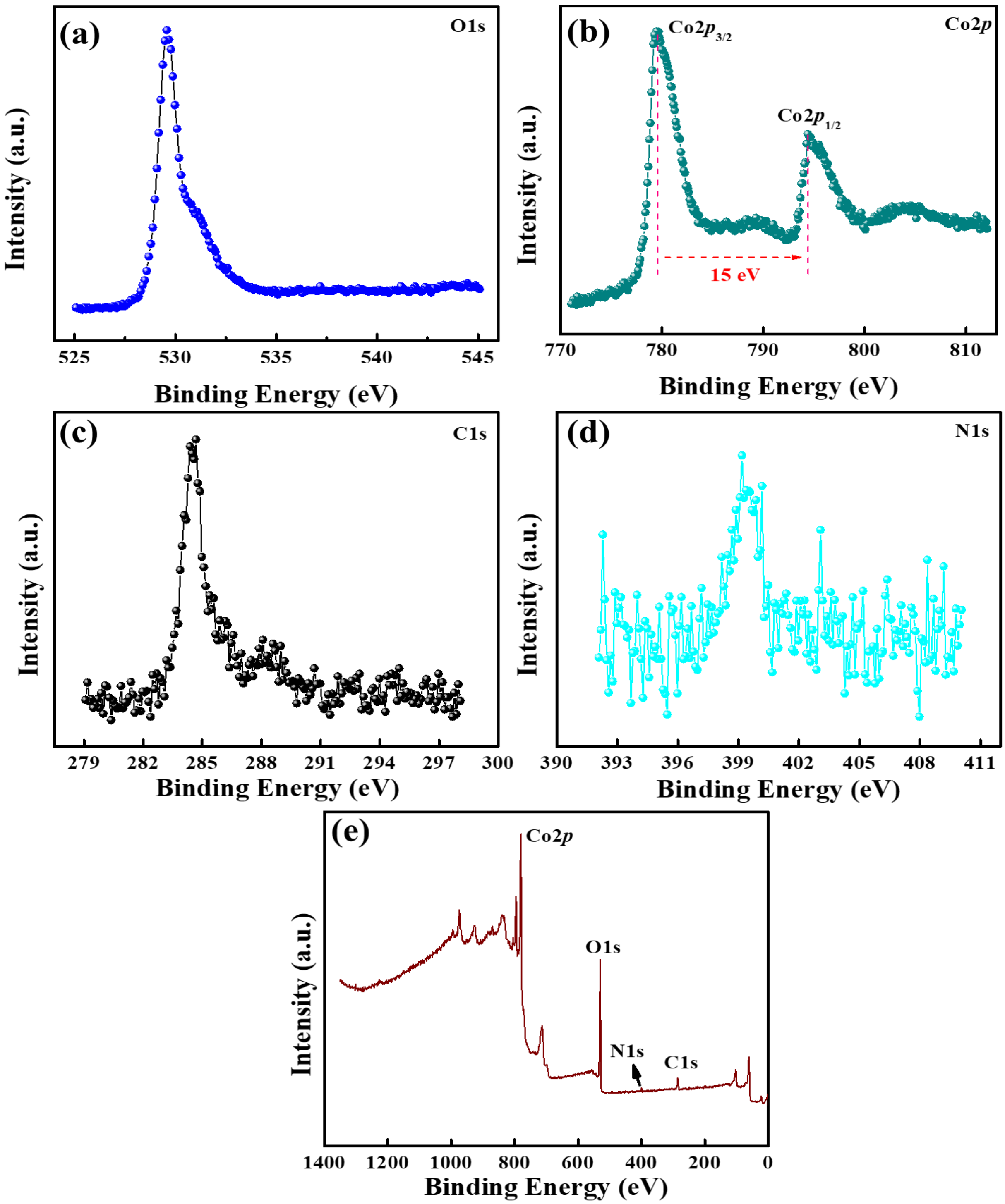

Figure S2. XPS analysis of Co_3O_4. (a) O1s, (b) Co2p, (c) C1s, and (d) N1s, and (e) survey spectrum.

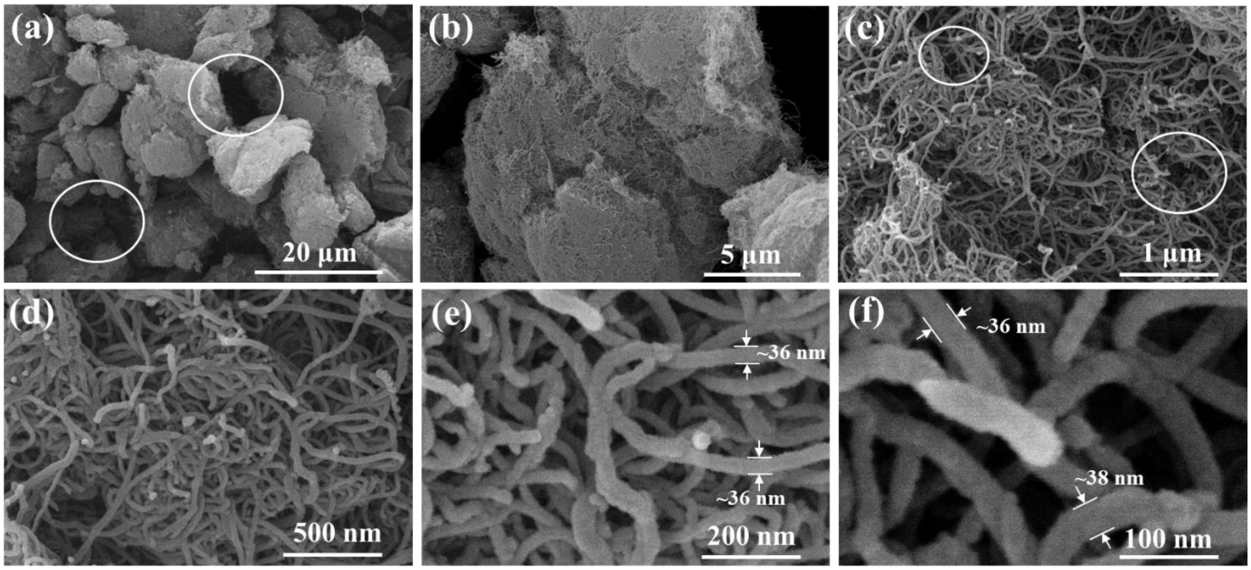

Figure S3. (a-f) FE-SEM images of the N-MWCNT at different magnifications.

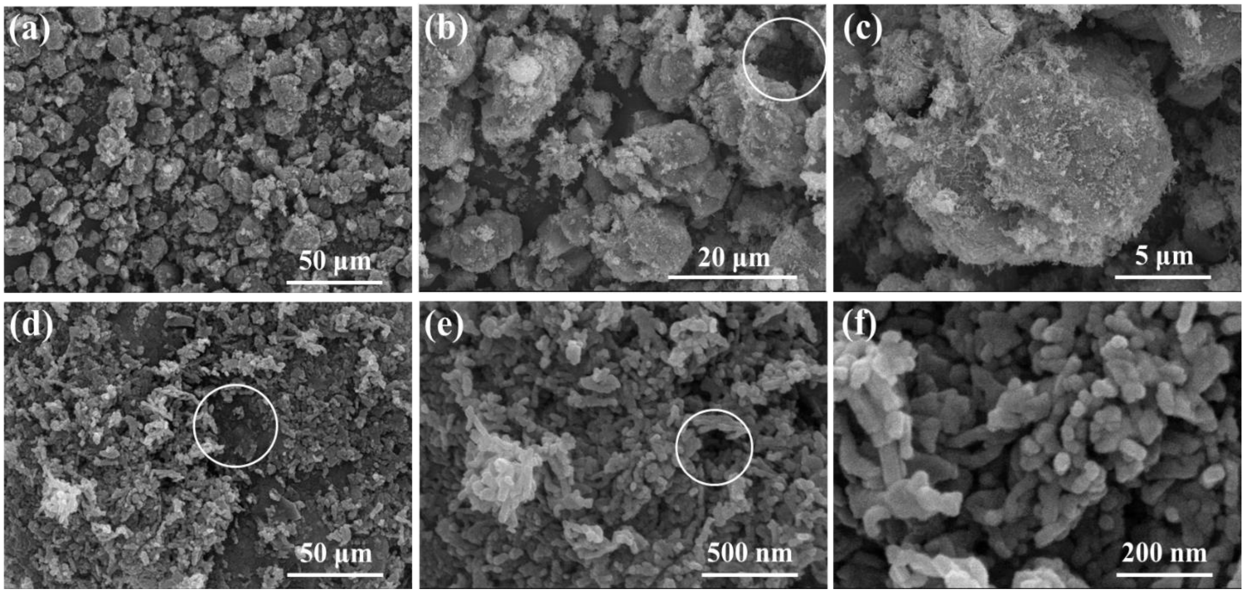

Figure S4. (a-f) FE-SEM images of Co_3O_4 at different magnifications.

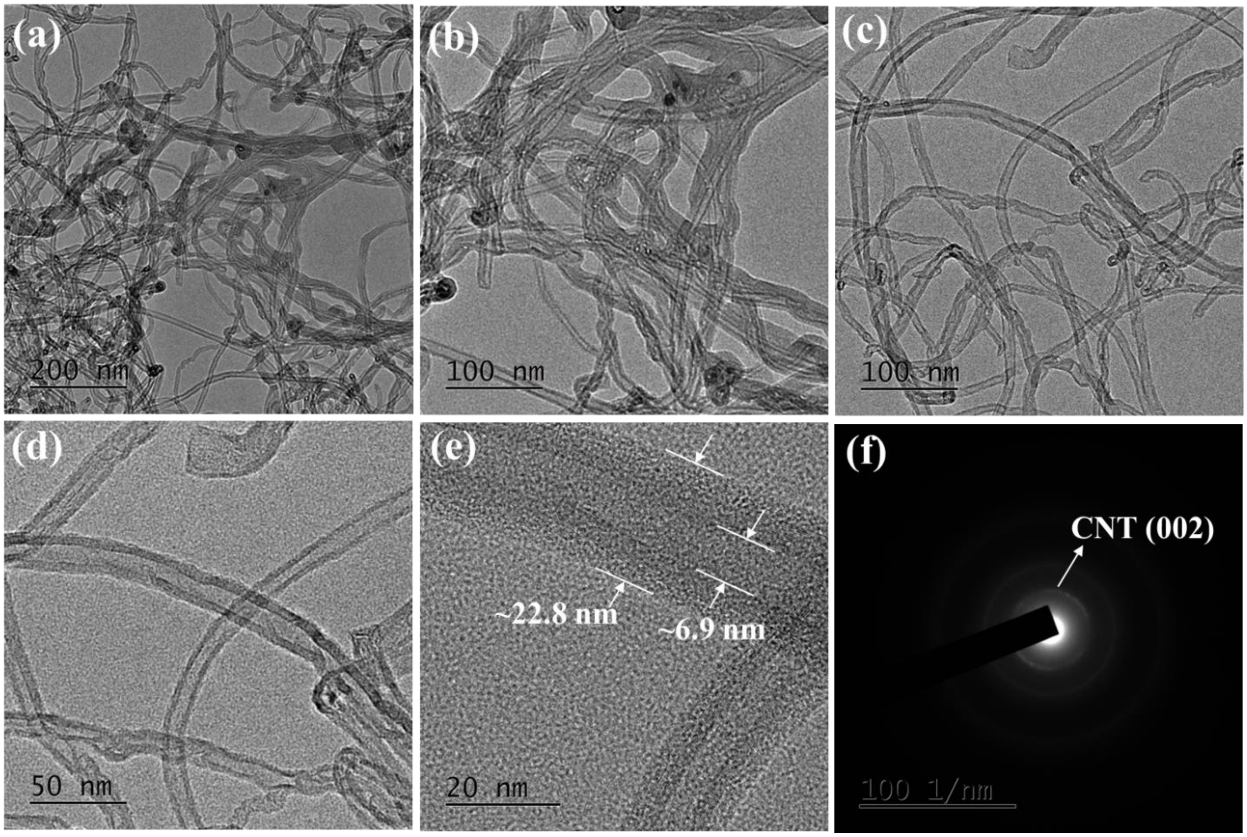

Figure S5. (a-e) FE-TEM images of N-MWCNT at different magnifications (f) SAED pattern of N-MWCNT.

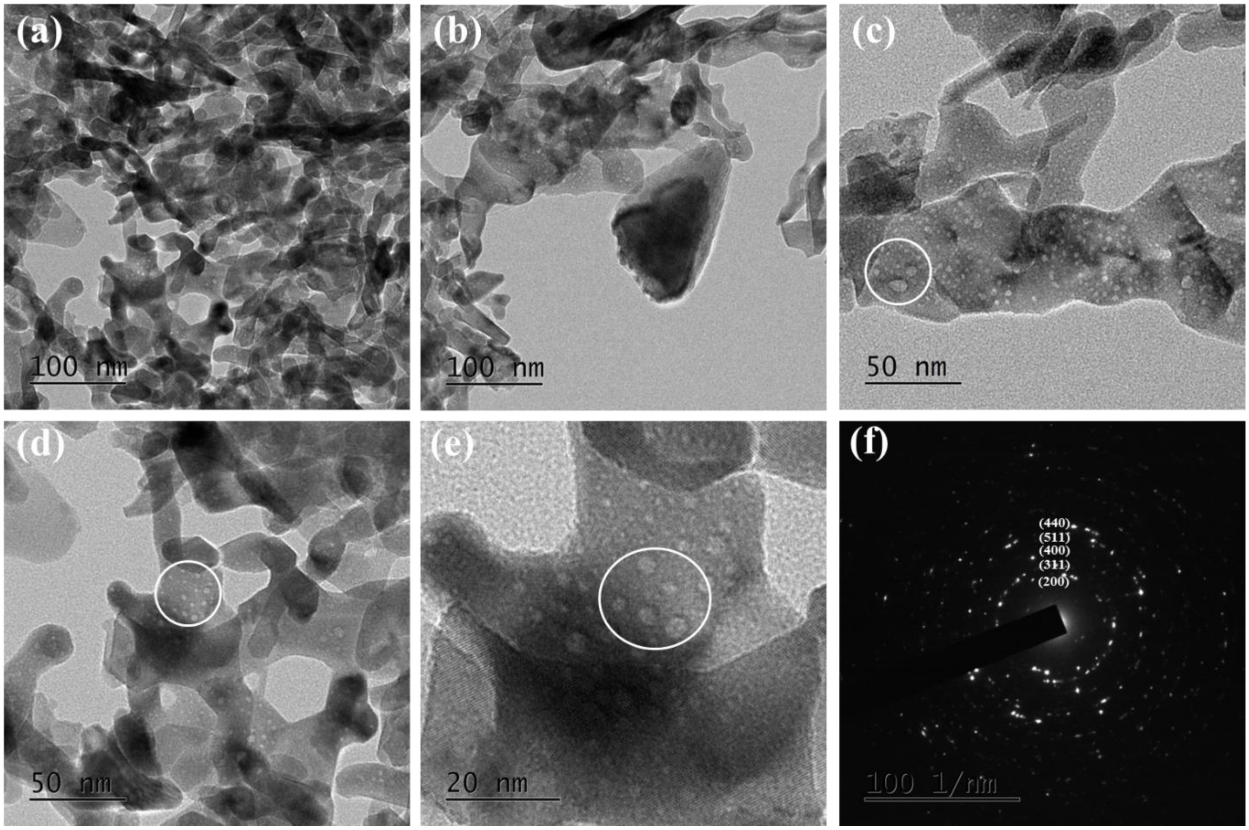

Figure S6. (a-e) FE-TEM images of Co_3O_4 at different magnifications and (f) SAED pattern of Co_3O_4.

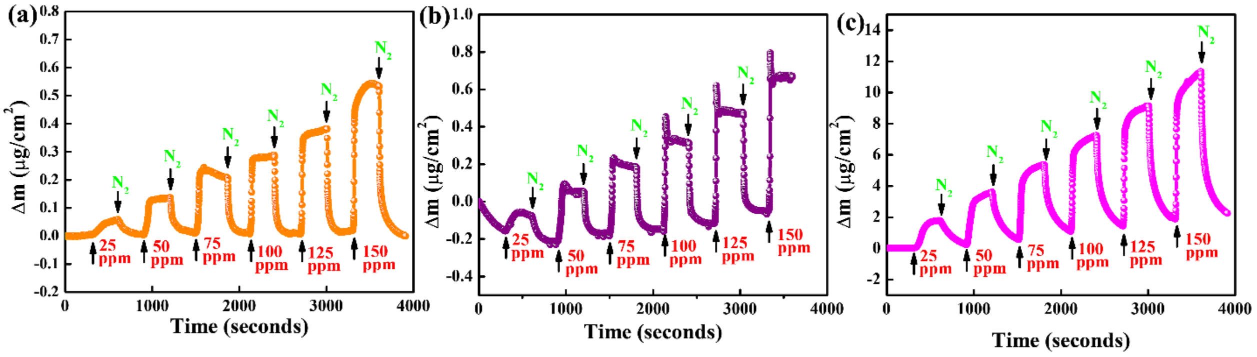

Figure S7. Mass of DMMP adsorbed by (a) Co_3O_4 (b) N-MWCNT, and (c) N-MWCNT@Co_3O_4 for DMMP concentrations ranging from 25 to 150 ppm.

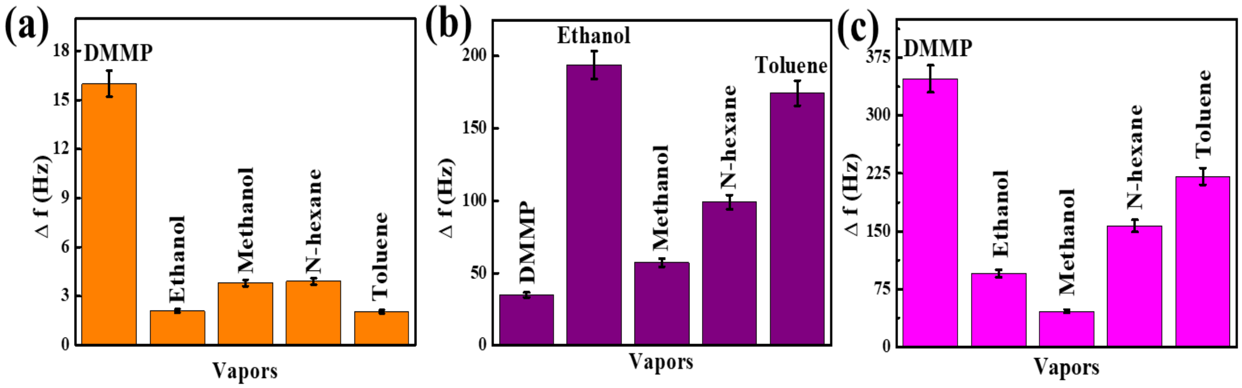

Figure S8. Selectivity of QCM sensors coated with (a) Co_3O_4, (b) N-MWCNT, (c) N-MWCNT@Co_3O_4 at a fixed flow rate (F_c = 200 sccm and F_d = 2000 sccm).

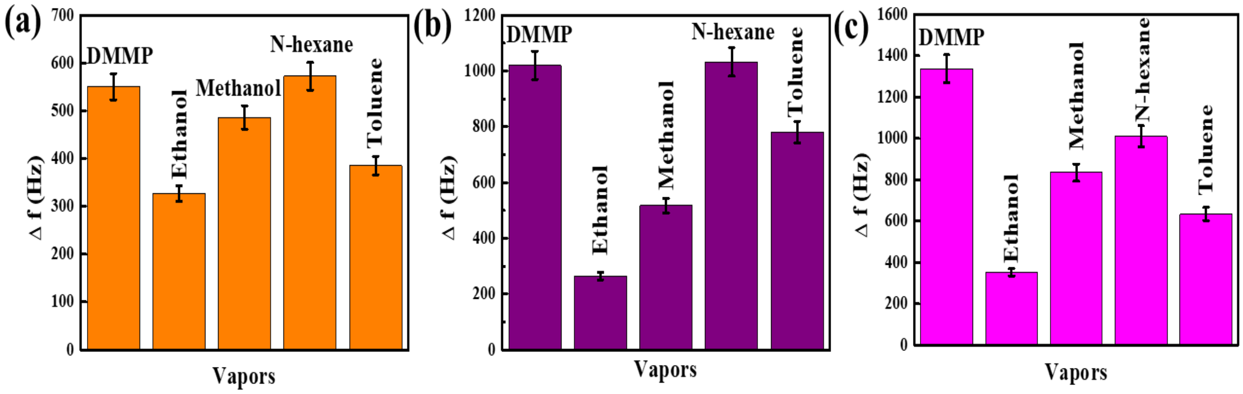

Figure S9. Selectivity of SAW sensors coated with (a) Co_3O_4, (b) N-MWCNT, (c) N-MWCNT@Co_3O_4 at a fixed flow rate (F_c = 100 sccm and F_d = 1000 sccm).

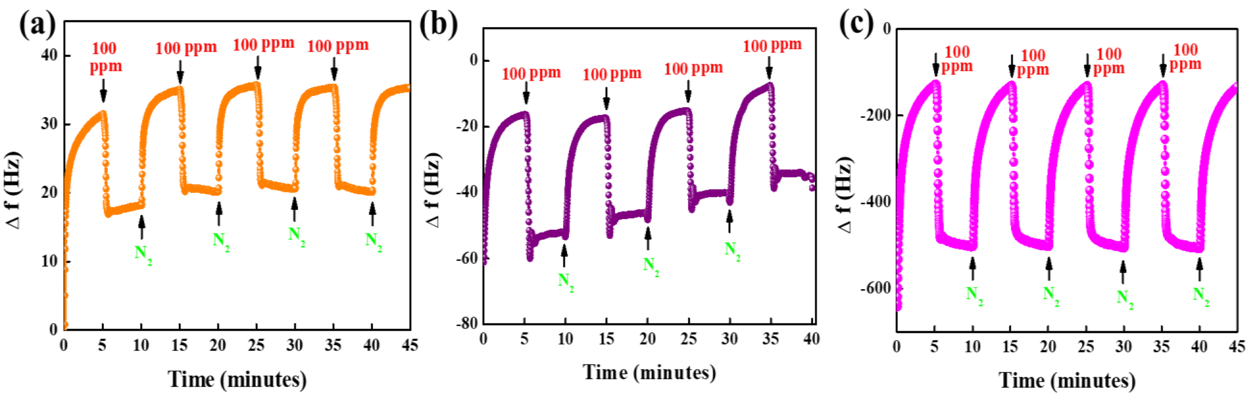

Figure S10. Repeatability of QCM sensors coated with (a) Co_3O_4, (b) N-MWCNT, and (c) N-MWCNT@Co_3O_4 at a fixed concentration of 100 ppm DMMP vapor.

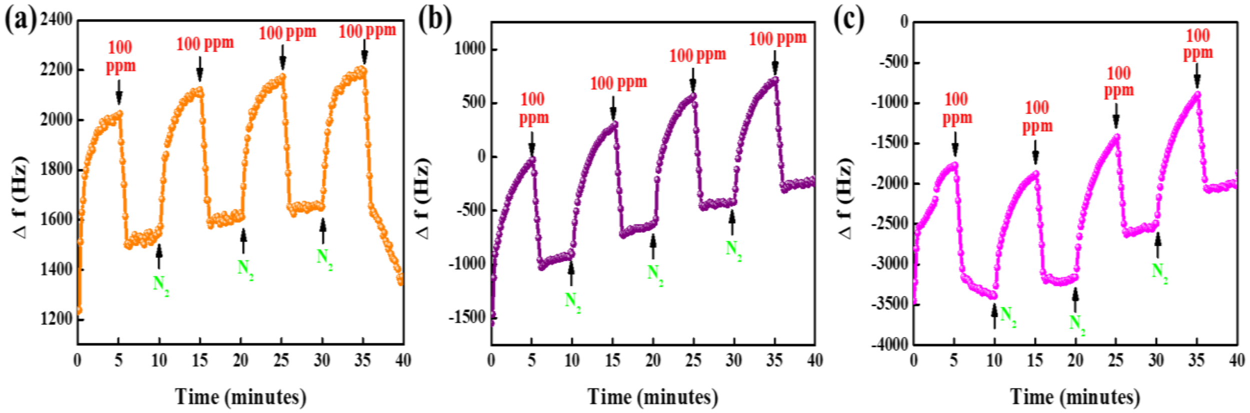

Figure S11. Repeatability of SAW sensors coated with (a) Co_3O_4 (b) N-MWCNT, and (c) N-MWCNT@Co_3O_4 at a fixed DMMP vapor concentration of 100 ppm.

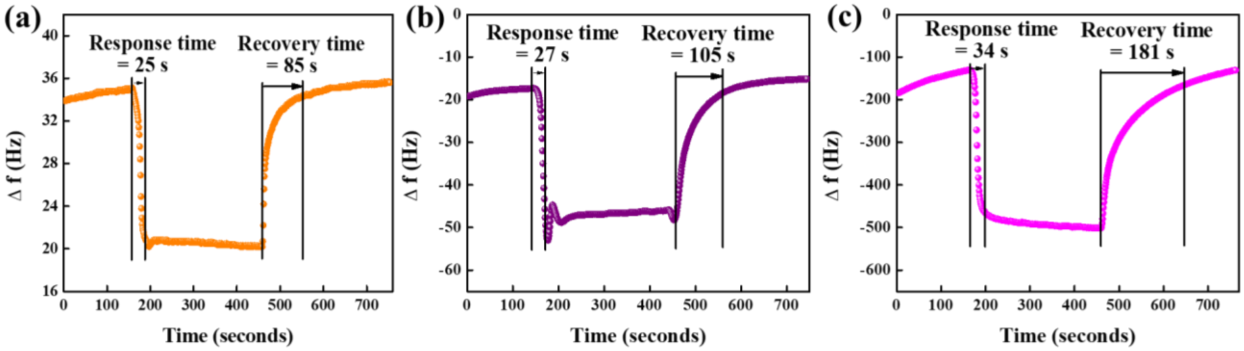

Figure S12. Response and recovery times of QCM sensors coated with (a) Co_3O_4, (b) N-MWCNT, and (c) N-MWCNT@Co_3O_4 at a fixed DMMP vapor concentration of 100 ppm.

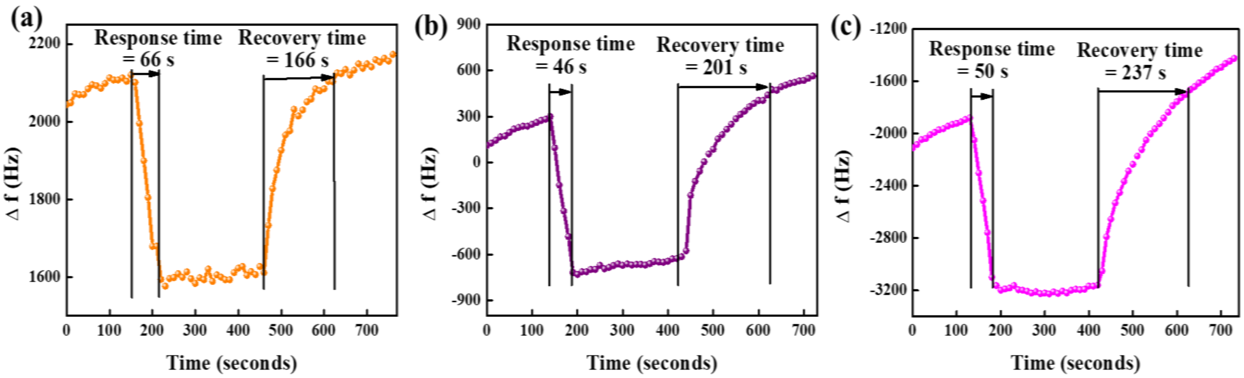

Figure S13. Response and recovery times of SAW sensors coated with (a) Co_3O_4, (b) N-MWCNT, and (c) N-MWCNT@Co_3O_4 at a fixed DMMP vapor concentration of 100 ppm.

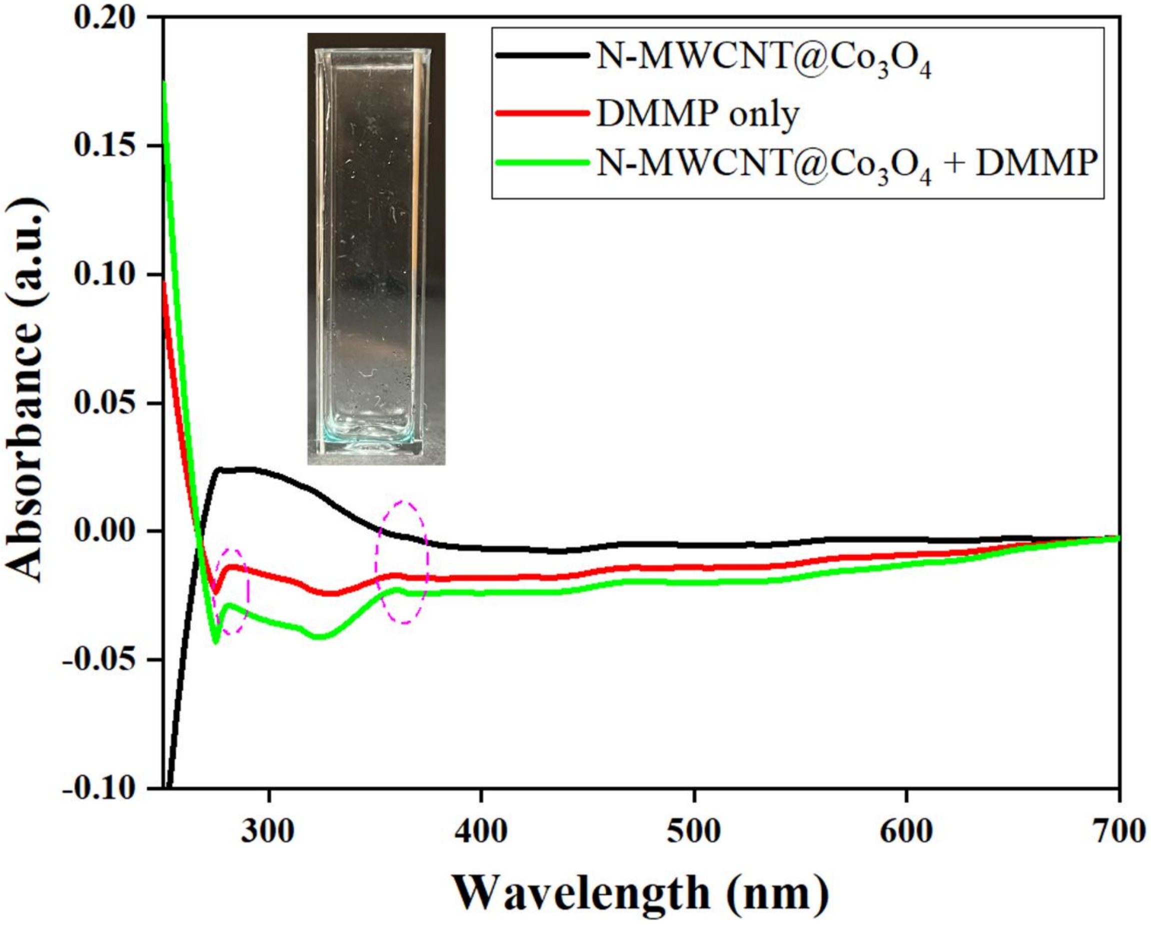

Figure S14. Absorbance spectra of N-MWCNT@Co_3O_4 only, DMMP only, and mixture of N-MWCNT@Co_3O_4 and DMMP.

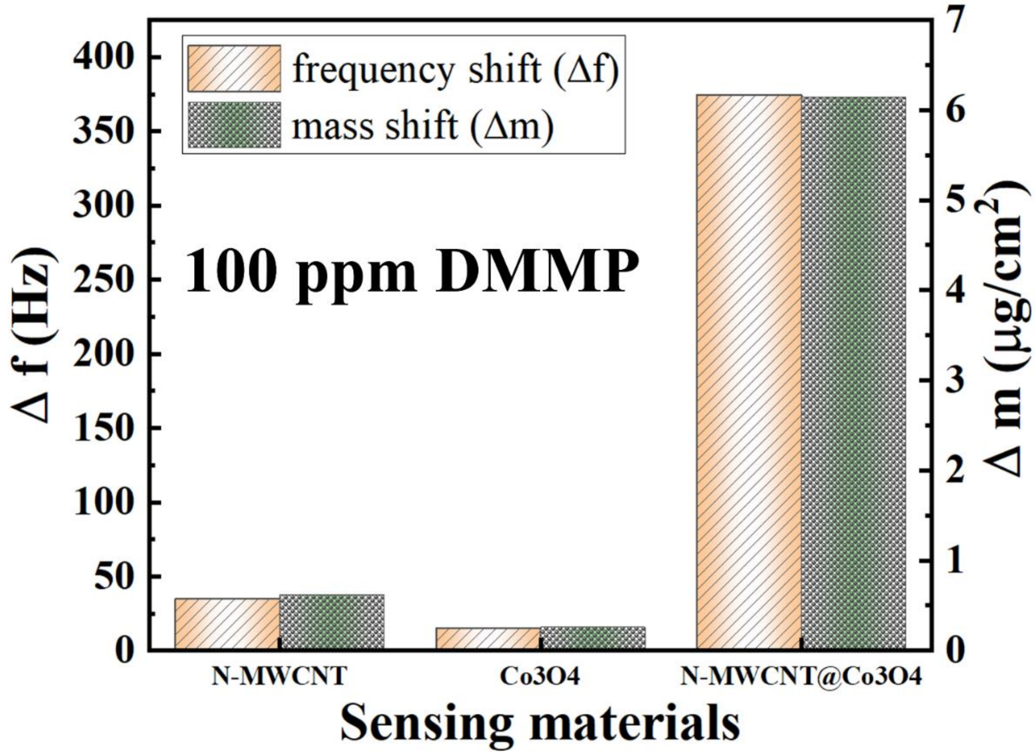

Figure S15. Comparison of frequency shift (Δ*f*) and mass shift (Δ*m*) of Co_3O_4, N-MWCNT, and N-MWCNT@Co_3O_4 at a fixed DMMP vapor concentration of 100 ppm.

References

[1] S. Lama, B. Bae, S. Ramesh, Y. Lee, N. Kim, J. Kim, Nano Sheet Like Morphology of Nitrogen Doped Graphene Oxide Grafted Manganese Oxide and Polypyrrole Composite for Chemical Warfare Agents Simulant Detection, Nanomaterials. 12 (2022) 2965.
